# Dissecting the psoriasis transcriptome: inflammatory- and cytokine-driven gene expression in lesions from 163 patients

**DOI:** 10.1186/1471-2164-14-527

**Published:** 2013-08-01

**Authors:** William R Swindell, Andrew Johnston, John J Voorhees, James T Elder, Johann E Gudjonsson

**Affiliations:** 1 Department of Dermatology, University of Michigan School of Medicine, Ann Arbor, MI 48109-2200, USA

**Keywords:** AP-1, Etanercept, IL-17, IL-20, Inflammation, Keratinocyte, Microarray, TNF, T-cell, Transcription factor

## Abstract

**Background:**

Psoriasis lesions are characterized by large-scale shifts in gene expression. Mechanisms that underlie differentially expressed genes (DEGs), however, are not completely understood. We analyzed existing datasets to evaluate genome-wide expression in lesions from 163 psoriasis patients. Our aims were to identify mechanisms that drive differential expression and to characterize heterogeneity among lesions in this large sample.

**Results:**

We identified 1233 psoriasis-increased DEGs and 977 psoriasis-decreased DEGs. Increased DEGs were attributed to keratinocyte activity (56%) and infiltration of lesions by T-cells (14%) and macrophages (11%). Decreased DEGs, in contrast, were associated with adipose tissue (63%), epidermis (14%) and dermis (4%). KC/epidermis DEGs were enriched for genes induced by IL-1, IL-17A and IL-20 family cytokines, and were also disproportionately associated with AP-1 binding sites. Among all patients, 50% exhibited a heightened inflammatory signature, with increased expression of genes expressed by T-cells, monocytes and dendritic cells. 66% of patients displayed an IFN-γ-strong signature, with increased expression of genes induced by IFN-γ in addition to several other cytokines (e.g., IL-1, IL-17A and TNF). We show that such differences in gene expression can be used to differentiate between etanercept responders and non-responders.

**Conclusions:**

Psoriasis DEGs are partly explained by shifts in the cellular composition of psoriasis lesions. Epidermal DEGs, however, may be driven by the activity of AP-1 and cellular responses to IL-1, IL-17A and IL-20 family cytokines. Among patients, we uncovered a range of inflammatory- and cytokine-associated gene expression patterns. Such patterns may provide biomarkers for predicting individual responses to biologic therapy.

## Background

Psoriasis is a chronic disease affecting 1-2% of the adult population, with estimates rising as high as 8% in certain geographic regions [1]. Psoriasis plaque formation is largely driven by cytokine-mediated interactions among dendritic cells, T-cells and keratinocytes (KCs), leading to altered differentiation and extensive KC proliferation [2,3]. To better understand this process, genome-wide expression profiling has been used to identify genes and pathways altered in psoriasis lesions as compared to normal skin [4-10]. Over the years, the power of this approach has improved as studies have scaled up to include samples from more patients, increasing statistical power to generate more robust findings. Two independent microarray studies, for instance, have now been performed using large cohorts with more than 60 patients each [8,11]. These data have allowed investigators to robustly identify differentially expressed genes and extract gene lists [4-10]. However, while individual genes with altered expression have been identified, underlying mechanisms remain unclear. Further work is therefore needed to understand how expression patterns in psoriasis lesions are connected to the inflammatory and cytokine dynamics that drive plaque formation.

Large-scale alteration of gene expression in psoriasis plaques is driven, in part, by differences in the composition and abundance of cell types present within lesional and non-lesional skin [6,12]. KC proliferation, for instance, is a hallmark of psoriasis lesions [2,3], and consequently psoriasis-increased genes include those genes expressed at high levels in KCs [6,12]. Likewise, psoriasis plaque formation is associated with an influx of non-resident immune cells, including T-cells, dendritic cells, macrophages and neutrophils [2,3], leading to the formation of dermal/epidermal inflammatory cell aggregates [13], and thus to increased expression of genes specifically expressed in lymphocytes and myeloid-derived cells. In psoriasis [6], and other conditions [14,15], these tissue remodeling processes have been investigated using statistical approaches applied to genome-wide expression patterns, which identify “signature genes” associated with individual cell types, and apply this information to identify shifts in cellular composition and to quantify the magnitude of these shifts. This approach, while informative, is not itself sufficient to understand the psoriasis transcriptome, however, since altered expression may also result from the activation or inhibition of cytokine-responsive pathways in resident cell types [5,6,16]. In cultured KCs, for example, stimulation with IL-17A leads to induction of β-defensins and pro-inflammatory S100 proteins [17], and such genes are consistently increased in psoriasis lesions [5,8-10]. In psoriasis, such cytokine-driven transcriptional responses of resident cells are superimposed upon shifts in cellular composition, adding a second layer of complexity to the transcriptome. Here again, however, statistical approaches have proven effective, and prior work has identified “cytokine activity signatures” embedded within the psoriasis transcriptome, based upon comparison of genes altered in psoriasis lesions with those altered in KCs stimulated by specific cytokines *in vitro*[5,6,16,18].

A second important, but less well explored, aspect of the psoriasis transcriptome is the heterogeneity observed among lesions sampled from different patients [6,7]. Previous studies have often focused on the expression of genes showing consistent differences between lesional and non-lesional skin, emphasizing analysis of differentially expressed genes (DEGs) [8-10]. While understanding mechanisms that drive differential expression remains important, the advent of larger datasets now permits new questions to be addressed [6,7]. One study, for instance, has identified two molecular sub-types of psoriasis based upon paired lesional (PP) and non-lesional (PN) samples from 37 patients, with one group (21 patients) characterized by PP-elevated expression of *HLA-E*, and the other group (16 patients) by elevated expression of genes involved in adaptive immunity (e.g., *CTLA-4*, *IFI30*, *IL4IL*, *PTPN2* and *SERPINB8*) [7]. Another study, with 62 patients, divided patients into three sub-groups based upon scores quantifying inflammatory infiltration by immune cell types (e.g., macrophages, dendritic cells and monocytes), along with two sub-groups differing in the expression of genes activated by IL-13 and other cytokines (e.g., IFN-α, TNF, IL-1a, IL-17A, and IFN-γ) [6]. With larger datasets, the finer-scale characterization of these variations should improve our ability to discern molecular sub-types. Ultimately, this may facilitate development of expression-based biomarkers that are biologically meaningful, but also useful for clinical applications (e.g., predicting response to biologic therapy) [6,7].

In this study, we analyzed genome-wide expression patterns in the involved (PP) and uninvolved (PN) skin from 163 psoriasis patients. Our analysis combines microarray data from three separate studies, each of which profiled gene expression using the same oligonucleotide array platform [8,11,19]. Using these data, we identify genes differentially expressed between PP and PN skin (i.e., DEGs), and assess whether these genes are enriched for cytokine-responsive genes or genes specifically expressed in distinct cell populations. Based upon these results, we associate most DEGs with a specific cell type and/or cytokine, and we have further identified mechanisms of transcriptional regulation by testing for association with transcription factor binding sites. Finally, we mapped the lesion-to-lesion variation associated with distinct inflammatory and cytokine signatures, leading to the identification of molecular sub-types among the 163 lesions. Extending these results, we show that such heterogeneous aspects may be associated with the response of patients to etanercept therapy.

## Results

### 80% of genes significantly elevated in psoriasis lesions can be explained by KC activity and infiltration by T-cells and macrophages

We assembled expression data from three studies in which raw data had been deposited in Gene Expression Omnibus (GSE13355, GSE14905 and GSE30999; Affymetrix Human Genome Plus 2.0 array; see Methods) [8,11,19]. Following quality control analyses, pooled data included paired lesional (PP) and non-lesional (PN) biopsies from 163 patients. For each patient, we calculated the difference (PP – PN) in expression for each probe set on the array. Based upon these differences, we identified 1233 differentially expressed genes (DEGs) with significantly increased expression in psoriasis lesions (FDR < 0.05 and FC > 1.50).

**Figure 1:**
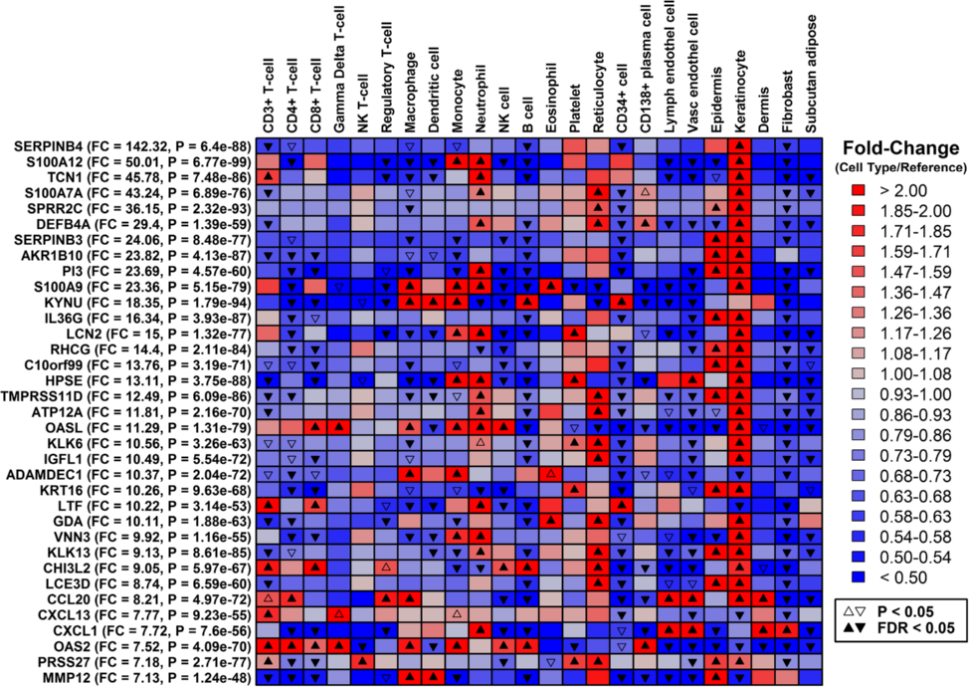
**Cell type-specific expression of the 35 genes most strongly elevated in psoriasis lesions (PP) compared to uninvolved skin (PN).** The left margin lists the 35 genes most strongly elevated in PP skin relative to PN skin (FDR < 0.05; ranked according to PP/PN fold-change). Heatmap colors show fold-change estimates for each of 24 cell types (columns), with fold-changes estimated as the ratio of a gene’s expression in a given cell type (numerator), relative to its expression among the 23 other cell types (denominator). Triangle symbols denote cases in which gene expression is significantly altered in one cell type as compared to all other cell types (see legend).

**Figure 2:**
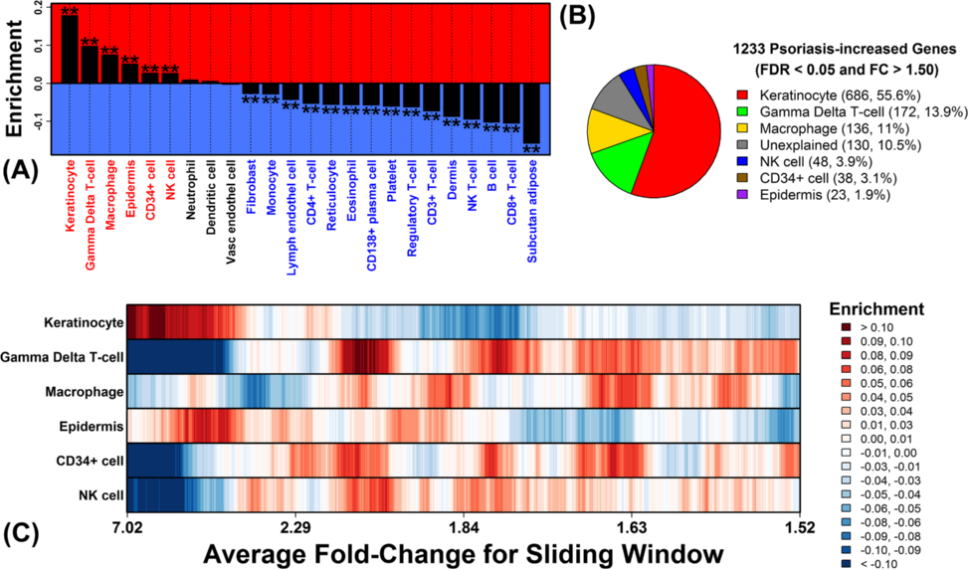
**Genes significantly elevated in psoriasis lesions can be explained by KC activity and infiltration of lesions by T-cells and macrophages. (A)** The 1233 PP-increased genes were analyzed to assess enrichment for genes specifically expressed in each of 24 cell types. Positive enrichment statistics indicate that PP-increased genes were more likely to be specifically expressed in that cell type relative to 18793 non-DEGs. Negative statistics indicate that PP-increased genes were less likely to be specifically expressed in that cell type relative to the 18793 non-DEGs. Asterisk symbols denote significantly large or small enrichment statistics (Wilcoxon rank sum test; FDR < 0.05). **(B)** The 1233 PP-increased genes were each assigned to one of the six cell types with significant and positive enrichment statistics in part **(A)**. A gene was assigned to a cell type if it was specifically expressed in that cell type (FDR < 0.05 and FC > 1.50). If a gene could be assigned to more than one cell type, it was assigned to the single cell type for which enrichment was highest in part **(A)**. The pie chart shows the proportion of 1233 PP-increased genes assigned to each cell type. The “unexplained” category includes PP-increased genes not specifically expressed in any of the six significant cell types. **(C)** The 1233 PP-increased genes were ranked according to fold-change increase in PP versus PN skin. Windows of 100 genes each were then evaluated at each point in the ranking to assess enrichment for genes specifically expressed in a given cell type (see legend).

We hypothesized that some PP-increased DEGs could be explained by KC activity and increased abundance of inflammatory cell types within lesions (e.g., T-cells and macrophages). To assess this possibility, we assembled a microarray database containing samples associated with a diverse range of 24 cell types (see Methods). Using this database, we identified genes showing a cell type-specific expression pattern*, with expression significantly higher in samples for one cell type, as compared to the other 23 cell types. As expected, DEGs most strongly increased in PP skin (e.g., *SERPINB4*, *S100A12* and *TCN1*) showed a KC-specific expression pattern, with significantly higher expression in KCs as compared to other cell types (Figure 1). We next tested whether the 1233 PP-increased DEGs overlapped significantly with genes specifically expressed in skin-associated cell types, including resident cells (e.g., KCs, fibroblasts and adipocytes) and potentially infiltrating inflammatory cells (e.g., T-cells, monocytes, macrophages and dendritic cells). This identified six cell types for which cell type-specific genes were enriched among the 1233 PP-increased DEGs, including KCs, γδ T-cells, macrophages, epidermis, CD34+ cells and NK cells (FDR < 0.05; Figure 2A). [*Note: By “cell type-specific” expression, we refer to genes with quantitatively higher expression in one specific cell type, even though such genes might have detectable expression in multiple cell types].

What fraction of PP-increased DEGs can be explained by the six cell types we identified? To address this question, we evaluated each DEG individually, and assigned each DEG to a cell type if the gene was specifically expressed in that cell type (FDR < 0.05 and FC > 1.50). If a DEG could be assigned to more than one cell type, it was preferentially assigned to the single cell type for which cell type-specific genes were most strongly enriched among all PP-increased DEGs (Figure 2A). Overall, 90% of the DEGs could be assigned to the six cell types, with most (80%) attributed to KCs (56%), γδ T-cells (14%) and macrophages (11%) (Figure 2B). DEGs assigned to KCs, for instance, included *SERPINB4*, *S100A12*, *TCN1* and *KRT16*, while those assigned to γδ T-cells included *CD3G*, *CD3D*, *IFNG* and *TNIP3*. Closer inspection revealed further trends among the PP-increased DEGs. In particular, DEGs most strongly elevated in PP skin (FC > 3) were enriched for genes highly expressed in KCs and epidermis (Figure 2C). However, DEGs less strongly elevated in PP skin (1.5 < FC < 3.0) were more highly expressed in inflammatory cells, such as γδ T-cells, macrophages and NK cells (Figure 2C).

These trends were reinforced by analysis of LCM-dissected dermal inflammatory cells from PP skin and LCM-dissected dermis from PN skin (*n* = 3 patients) [13]. We identified 609 genes elevated in LCM-dissected dermal inflammatory cells from PP skin (relative to LCM-dissected PN dermis; P < 0.05 and FC > 1.50). As expected, these genes were not significantly enriched for genes specifically expressed in KCs (Additional file 1, Part A). However, there was significant enrichment for genes specifically expressed in the three inflammatory cell types identified above (γδ T-cells, macrophages and NK cells) (FDR < 0.05; Additional file 1). Additionally, there was significant enrichment for other T-cell subsets, including CD3+ T-cells, CD4+ T-cells and CD8+ T-cells, regulatory T-cells and dendritic cells, suggesting that LCM may enhance the resolution for detection of expression shifts arising from the formation of immune cell aggregates in PP skin (Additional file 1, Part A). Using the same criteria stated above, we could assign a cell type to more than 90% of the 609 genes elevated in LCM-dissected dermal inflammatory cells from PP skin (Additional file 1, Part B).

### 80% of genes decreased in psoriasis lesions are specifically expressed in subcutaneous adipose tissue, dermis and epidermis

**Figure 3:**
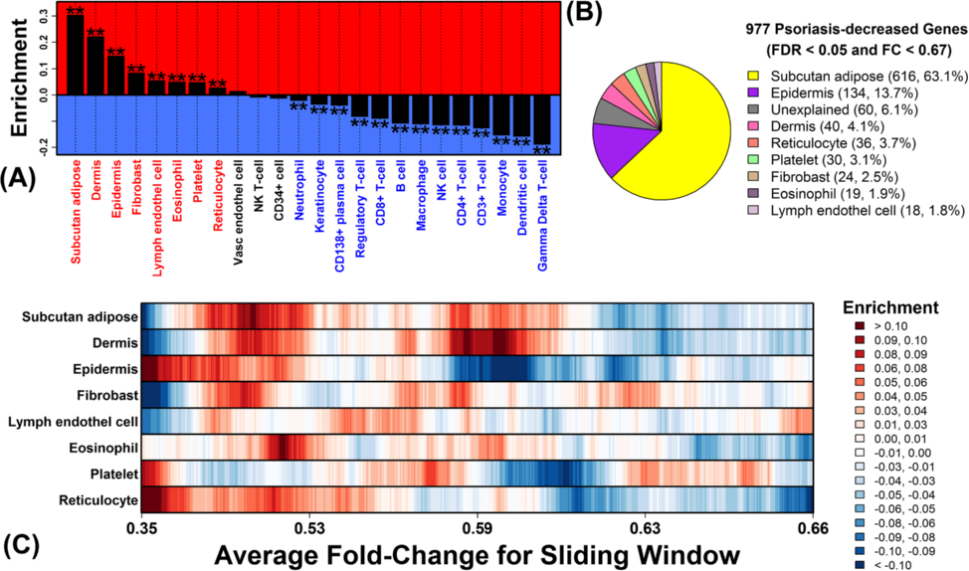
**80% of genes decreased in psoriasis lesions are specifically expressed in subcutaneous adipose tissue, dermis and epidermis. (A)** The 977 PP-decreased genes were analyzed to assess enrichment for genes specifically expressed in each of 24 cell types. Asterisk symbols denote significantly large or small enrichment statistics (Wilcoxon rank sum test; FDR < 0.05). **(B)** The 977 PP-decreased genes were each assigned to one of the eight cell types with significant and positive enrichment statistics in part **(A)**. A gene was assigned to a cell type if it was specifically expressed in that cell type (FDR < 0.05 and FC > 1.50). If a gene could be assigned to more than one cell type, it was assigned to the single cell type for which enrichment was highest in part **(A)**. The pie chart shows the proportion of 977 PP-decreased genes assigned to each cell type. **(C)** The 977 PP-decreased genes were ranked according to fold-change decrease in PP versus PN skin. Windows of 100 genes each were evaluated at each point in the ranking to assess enrichment for genes specifically expressed in a given cell type (see legend).

Our analysis of PP and PN samples from 163 patients identified 977 PP-decreased DEGs (FDR < 0.05 and FC < 0.67). Among genes most strongly decreased in PP skin (e.g., *BTC*, *WIF1* and *THRSP*), most were weakly expressed in myeloid-derived cell types, but did show specific expression in epidermis (Additional file 2). Among all 977 PP-decreased DEGs, we identified significant enrichment for genes specifically expressed in eight cell types (FDR < 0.05; Figure 3). Most of these were skin-resident cell types and in fact PP-decreased DEGs were most enriched for genes expressed at high levels in subcutaneous adipose tissue, dermis and epidermis (Figure 3A). To determine the proportion of PP-decreased DEGs that may be accounted for by these cell types, PP-decreased DEGs were inspected one-by-one, and each was assigned to one of the eight significant cell types (Figure 3A). Overall, 90% of PP-decreased DEGs could be assigned to at least one of the eight cell types (FDR < 0.05 and FC > 1.50), with 80% assigned to subcutaneous adipose tissue (63%), epidermis (14%) or dermis (4%) (Figure 3B). Further inspection revealed that, although DEGs most strongly decreased in PP skin (FC < 0.50) tended to be expressed in epidermis (Figure 3C), those DEGs with moderately decreased expression (0.50 < FC < 0.66) were more commonly expressed at high levels in subcutaneous adipose tissue or dermis/fibroblasts (Figure 3C).

### Epidermal genes elevated in psoriasis lesions overlap best with genes induced by IL-1, IL-17 and IL-20 family cytokines in cultured KCs

Of 1233 PP-increased DEGs, we designated 709 as “epidermal”, based upon their high expression in KCs or epidermis as compared to 22 other cell types in our analysis (Figure 2A and 2B). Potentially, increased expression of these genes may be driven by the altered cytokine environment within psoriasis lesions [5,6,16]. We thus compiled data from 42 experiments in which genome-wide expression was evaluated in cultured monolayer KCs (or 3-D reconstituted epidermis) following cytokine treatment (Additional file 3). As expected, among the top 30 epidermal PP-increased DEGs, most were increased by at least one cytokine treatment, with several DEGs increased by multiple cytokines (e.g., *SERPINB4*, *S100A12*, *SPRR2C*, *DEFB4A*, *PI3*; Additional file 4).

**Figure 4:**
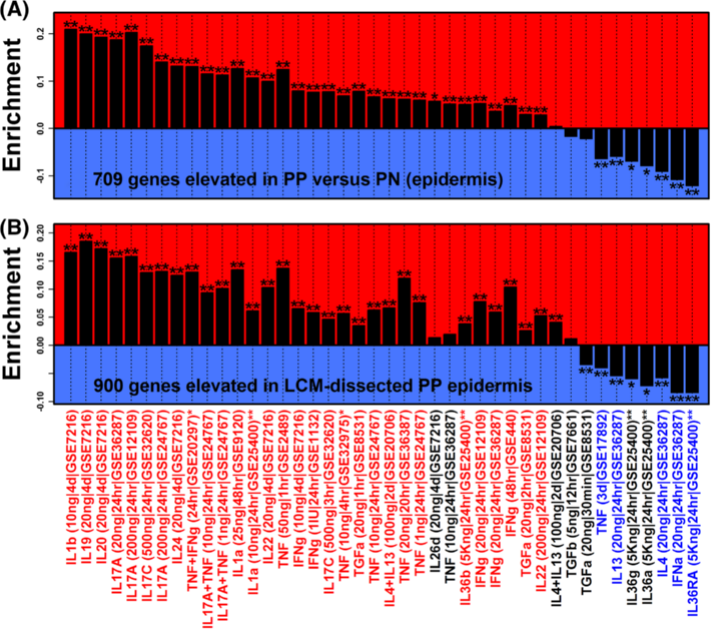
**Epidermal genes elevated in psoriasis lesions overlap best with genes induced by IL-1, IL-17, and IL-20 family cytokines in cultured KCs.** In part **(A)**, 709 epidermal PP-increased DEGs were evaluated to determine if they were disproportionately increased or decreased in each of 42 cytokine experiments. In each experiment, KCs or reconstituted epidermis was treated with cytokines and microarrays were used to identify induced genes. Experiments using HaCaT KCs are indicated with a single asterisk symbol (*), while experiments using 3-D reconstituted epidermis are indicated by a double asterisk (**). All other experiments utilized primary monolayer KC cultures. Labels list the cytokine used, the concentration (per mL), the length of time cells were treated, and the Gene Expression Omnibus accession under which raw data can be accessed. Positive statistics indicate that the 709 genes were disproportionately induced, while negative statistics indicate that the 709 genes were disproportionately repressed. A single asterisk symbol denotes significance according to the Wilcoxon rank sum test (FDR < 0.05), while two asterisk symbols denote significance according to both the Wilcoxon rank sum test and Fisher’s Exact Test (FDR < 0.05). In part **(B)**, the analysis was repeated based upon 900 genes significantly elevated in LCM-dissected lesional epidermis as compared to LCM-dissected epidermis from uninvolved skin (FDR < 0.05 & FC > 1.50). In the bottom margin, red labels denote experiments with significantly positive statistics in both **(A)** and **(B)** (FDR < 0.05). Blue labels denote experiments with significantly negative statistics in both **(A)** and **(B)** (FDR < 0.05).

We screened the 42 experiments to determine which cytokine treatments induced a set of genes that overlapped best with the 709 epidermal PP-increased DEGs. In parallel, we evaluated how well genes induced in each experiment overlapped with 900 genes significantly elevated in LCM-isolated epidermis from psoriasis lesions as compared to LCM-isolated epidermis from normal skin (FDR < 0.05 and FC > 1.50) [13]. We expected analysis of both gene sets to yield similar results, and indeed, there was good agreement, with significant and corresponding results obtained for 35 of the 42 experiments (Figure 4). Overall, three groups of cytokines induced a gene set that overlapped best with the 709 epidermal PP-increased DEGs, including the IL-1, IL-17 and IL-20 families (Figure 4). Of the 13 top-ranked experiments, nearly all (12 of 13) involved experiments in which cells were treated with a cytokine from one of these groups. Genes induced by IL-1, IL-17 and IL-20 family cytokines *in vitro* thus bear the closest resemblance to epidermal DEGs elevated in PP skin.

Among the 709 epidermal PP-increased DEGs, 143 (20%) were not significantly altered in any of the 35 experiments for which induced/repressed genes overlapped significantly with the 709 DEGs (e.g., *AKR1B10*, *C10orf99* and *WDR66*; Additional file 5). Such non-responsive DEGs may be increased in PP skin due to alteration in the KC differentiation program, or otherwise, may be elevated due to expansion of the KC compartment. Most DEGs (566 of 709), however, were significantly altered by cytokine treatment in at least one of the 35 significant experiments, and overall, we attributed the largest number of DEGs to induction by TNF + IFN-γ (174 DEGs), IL-1β (104 DEGs) and IL-17A (79 DEGs) (Additional file 5).

### Epidermal genes decreased in psoriasis lesions overlap best with genes repressed by IL-20 family cytokines in cultured KCs

DEGs most strongly decreased in psoriasis (FC < 0.50) were enriched for genes with an epidermis-specific expression pattern (Figure 3C), and overall, we classified 134 of the 977 PP-decreased DEGs as epidermal (Figure 3B). For some of these, expression was repressed *in vitro* following cytokine treatment of KCs or reconstituted epidermis (e.g., *KRT77*, *C5orf46* and *PLLP*; Additional file 6). We screened the 42 cytokine experiments (Additional file 3) to determine which repressed a set of genes that overlapped best with the 134 epidermal PP-decreased genes. In parallel, we evaluated how well genes repressed in each experiment overlapped with 876 genes significantly decreased in LCM-isolated epidermis from PP skin as compared to LCM-isolated epidermis from PN skin (FDR < 0.05 and FC < 0.67) [13]. We identified 10 experiments for which cytokine-repressed genes overlapped significantly with both sets of PP-decreased genes (Additional file 7). For two of these, cells were treated with IL-1β or IL-17A (Additional file 6), consistent with our findings for PP-increased genes (Figure 4). The strongest trends, however, were observed for cells treated with IL-10 family cytokines, including all three cytokines from the IL-20 family (IL-24, IL-19 and IL-20; Additional file 7). These results show that epidermal genes decreased in PP skin are most similar in composition to genes repressed by IL-10/IL-20 family cytokines *in vitro*. Overall, 80 of the 134 DEGs (60%) were significantly repressed in at least one of the 10 significant experiments, and we attributed more than half of these (46 DEGs) to repression by IL-22 (e.g., *KRT77*, *IL37* and *FABP7*; see Additional file 8).

### Enrichment of AP-1 binding sites among psoriasis-increased genes and evidence for activation of an IL-17A → AP-1 pathway

Our analysis uncovered 709 epidermal PP-increased DEGs (Figure 2), which were also enriched for genes induced by IL-1, IL-17 and IL-20 family cytokines (Figure 4). We hypothesized that some DEGs would be targeted by transcription factors (TFs) belonging to cytokine-responsive pathways. To address this possibility, we generated a motif dictionary with 1209 binding sites for known TFs or DNA-binding complexes, and we screened these sites to determine which were most enriched in 2 KB regions upstream of the 709 PP-increased DEGs (see Methods).

**Figure 5:**
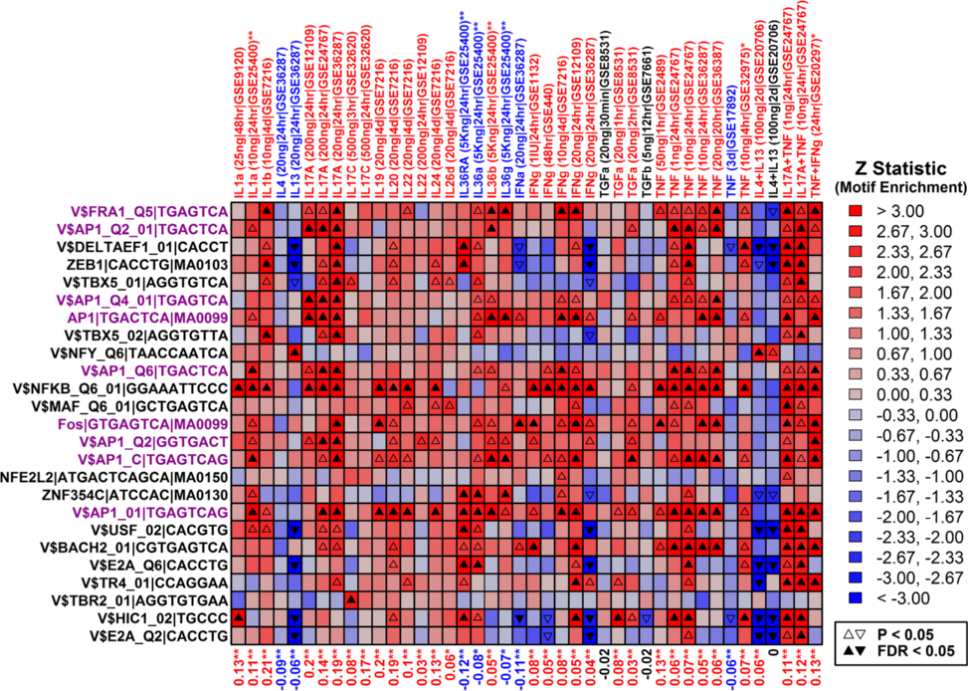
**Transcription factor binding sites enriched in 2KB regions upstream of 709 epidermal PP-increased DEGs.** We identified 709 epidermal PP-increased DEGs (FDR < 0.05 and FC > 1.50). For these genes, we scanned regions 2 KB upstream from the transcription start site for matches to 1209 transcription factor binding sites (see Methods). 27 binding sites were significantly enriched among the 709 genes (*Z* > 0 and FDR < 0.05; left margin). For these motifs (left margin), labels indicate the consensus binding site along with identifier information from the source database (UniPROBE, Jaspar or TRANSFAC). Magenta labels denote motifs recognized by the AP-1 complex. For each site, we tested whether its frequency was significantly elevated in upstream regions of genes induced by cytokines in each of 42 experiments (P < 0.05 and FC > 1.50). Heatmap colors show *Z* statistics from these analyses, where positive values indicate enrichment of a site in regions upstream of cytokine-induced genes, while negative values denote underrepresentation of a site in regions upstream of cytokine-induced genes. Significantly positive or negative Z statistics are denoted by triangle symbols (see legend). Values in the bottom margin list enrichment statistics that assess whether the 709 PP-increased DEGs are disproportionately elevated or repressed in the cytokine experiment (see Figure 4A). Red labels denote significantly positive statistics, indicating that the 709 PP-increased DEGs are disproportionately induced in a given cytokine experiment (Wilcoxon rank sum test and Fisher’s Exact Test; FDR < 0.05). Blue labels denote significantly negative statistics, indicating that the 709 PP-increased DEGs are disproportionately repressed in an experiment (Wilcoxon rank sum test and Fisher’s Exact Test; FDR < 0.05).

We identified 27 sites enriched among the 709 DEGs, including 9 associated with AP-1 (FDR < 0.05; Figure 5). To confirm this trend, we repeated the analysis starting with 900 genes elevated in LCM-dissected epidermis (Additional file 9). Based on this gene set, we did not identify sites significant at an FDR threshold of 0.05. However, 5 of the 25 sites most enriched among the 900 genes were associated with the AP-1 complex (P ≤ 0.014; FDR ≤ 0.49) (Additional file 9). Further analysis revealed that genes encoding AP-1 components were differentially expressed in psoriasis lesions (Additional file 10). Among the 163 patients, there was significantly increased expression of *JUNB*, *FOSL1* and *FOSB* in PP skin, along with significantly decreased expression of *JUND*, *FOSL2* and *FOS* (Additional file 10, Part A). Expression of *JUNB, FOSL1* and *FOSL2*, moreover, was significantly elevated in LCM-dissected epidermis from PP skin (Additional file 10, Part B), while expression of *JUNB* and *FOSL2* was also elevated in LCM-dissected dermis from PP skin (Additional file 10, Part C).

We next analyzed the 27 significant sites to determine if they were also enriched in 2 KB regions upstream of cytokine-induced genes (Figure 5). Not surprisingly, an NF-κB site was enriched in regions upstream of the 709 epidermal PP-increased DEGs, as well as in regions upstream of genes induced by IL-1- and IL-20-family cytokines, IL-17A, IFN-γ and TNF (Figure 5). Interestingly, however, among the 9 AP-1 sites enriched in regions upstream of PP-increased genes, each was also enriched in regions upstream of genes induced by IL-17A, and this result was replicated in 2-3 independent experiments (GSE12109, GSE24767 and GSE36287; Figure 5). These results are consistent with activation of an IL-17A → AP-1 pathway in PP skin.

### Psoriasis lesions from 163 patients can be divided into two sub-groups based upon inflammatory gene expression patterns (strong inflammation: 89/163; weak inflammation: 74/163)

**Figure 6:**
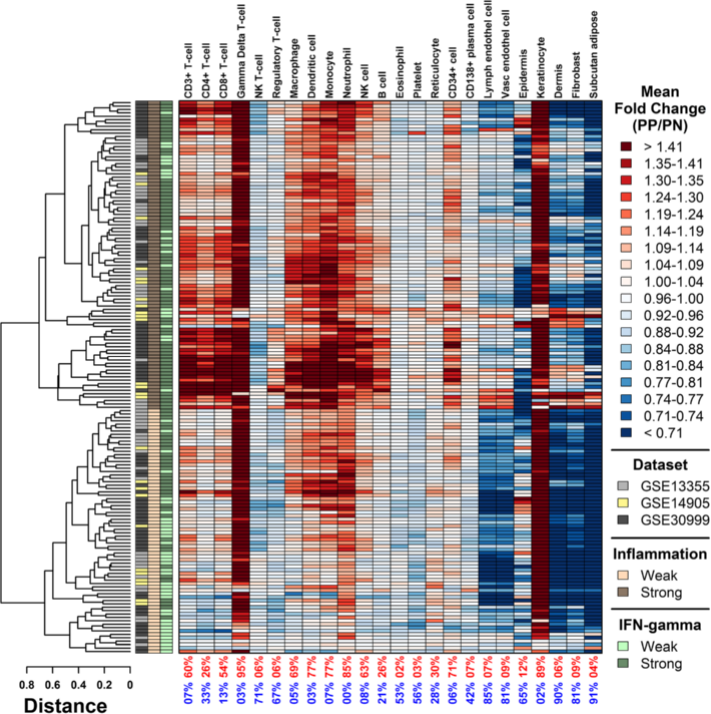
**Psoriasis lesions from 163 patients can be divided into two subgroups based upon inflammatory gene expression patterns (strong inflammation: 91/163; weak inflammation: 72/163).** For each cell population, we identified 250 “signature genes” most specifically expressed in that cell population relative to all other cell types. For each patient, a signature score was calculated as the weighted average of fold-changes (PP/PN) among these 250 signature genes (weighted arithmetic mean). Weighted averages were calculated by assigning greatest weight to the top-ranked gene most specifically expressed in a given cell type, with weights declining between the top-ranked and 250th-ranked gene. Values in the bottom margin indicate the proportion of patients with significantly large (red) or small (blue) signature scores (P < 0.05). Significance was evaluated by comparing fold-changes (PP/PN) of the 250 signature genes to those of all other genes represented on the array (Wilcoxon rank sum test). Patients were clustered according to signature scores using complete linkage and the Euclidean distance metric. Group assignments were made based upon whether a patient’s scores approximated a strong or weak pattern of inflammation (see legend).

Gene expression patterns vary in direction and magnitude among lesions from different psoriasis patients, potentially reflecting distinct molecular-level sub-types [6,7]. For the 163 patients, we calculated signatures corresponding to inflammatory and skin-resident cell types, where the value of each signature is equal to the weighted average of fold-changes (PP/PN) among the 250 genes most specifically expressed in that cell type. Consistent with our analysis of DEGs (Figure 2), lesions from nearly all patients (≥ 89%) were associated with significantly large γδ T-cell and KC signatures (Figure 6). Other inflammatory signatures were less consistent among patients (Figure 6). Using cluster analysis, we identified 91 patients (56%) with a strong inflammatory signature, characterized by heightened expression of genes specifically expressed in CD3+ T-cells, CD4+ T-cells, CD8+ T-cells, macrophages, dendritic cells, monocytes, neutrophils, NK cells and B cells (Figure 6). The remaining 72 patients (44%) were associated with a weak inflammatory signature, with reduced expression of genes specifically expressed in these cell types (Figure 6).

### Psoriasis lesions from 163 patients can be divided into two sub-groups based upon the expression of cytokine-induced genes (IFN-γ-strong: 104/163; IFN-γ-weak: 59/163)

**Figure 7:**
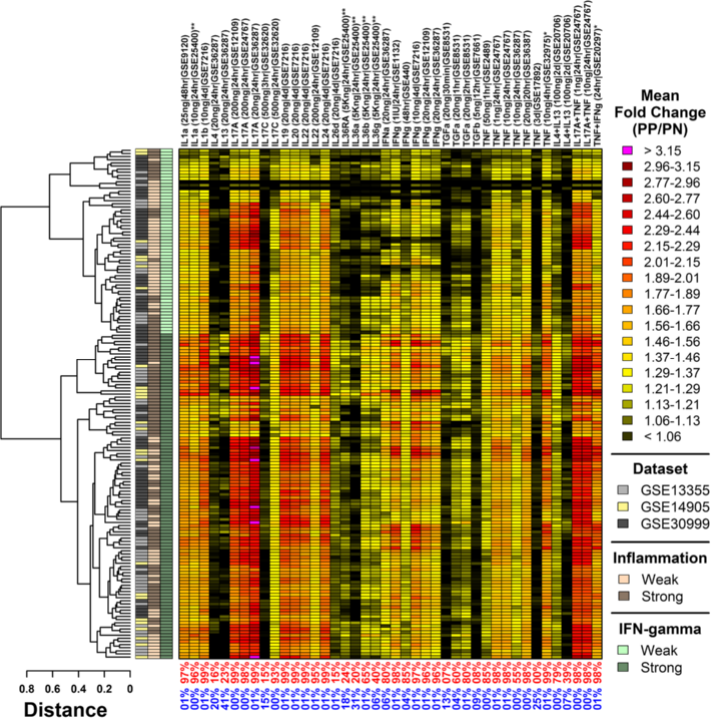
**Psoriasis lesions from 163 patients can be divided into two subgroups based upon the expression of cytokine-induced genes (IFN-γ-strong: 104/163; IFN-γ-weak: 59/163).** Signature scores were calculated for each patient based upon the 250 genes most strongly induced in each of 42 cytokine experiments (top margin). Signature scores were calculated as the weighted average of fold-changes (PP/PN) among the 250 most strongly induced genes (weighted arithmetic mean). The greatest weight was assigned to the top-ranked gene, with weights declining between the top-ranked and 250th-ranked gene. Values in the bottom margin indicate the proportion of patients for which signature scores were significantly large (red) or small (blue). Significance was evaluated by comparing fold-changes (PP/PN) of the 250 signature genes to those of all other genes represented on the array (Wilcoxon rank sum test). Patients were clustered according to signature scores using complete linkage and the Euclidean distance metric. Group assignments were made based upon whether a patient’s scores approximated a strong or weak pattern of IFN-γ activity (see legend).

We next calculated signature scores based upon genes induced in each of 42 cytokine experiments (Additional file 3), where the value of each score was equal to the weighted average of fold-changes estimated for the top 250 cytokine-induced genes (Figure 7). Signature scores calculated for several cytokines, including IL-1α and IL-17A, were significantly elevated in nearly every patient (≥ 99%; Figure 7). However, the magnitude of such effects varied, particularly with respect to IFN-γ, IL-1α, IL-17A, IL-22, IL-36β, IL-36γ and TNF (Figure 7). Using cluster analysis, we identified 104 patients (64%) with heightened scores for these cytokines, corresponding to elevated expression of cytokine-induced genes (Figure 7). The remaining 59 patients (36%) were associated with weaker scores and lower expression of cytokine-induced genes (Figure 7). We refer to these groups as IFN-γ-strong and IFN-γ-weak, respectively, since the distinction between groups was especially strong with respect to the five IFN-γ signatures included in our analysis (Figure 7). Notably, patients in the IFN-γ-weak group also tended to exhibit weak inflammatory patterns -- 63% those with IFN-γ-weak signatures were assigned to the weak inflammatory group (cf., 44% of all patients belonged to the weak inflammation group).

### Lesions obtained at baseline from etanercept responders show increased expression of TNF-induced genes and genes specifically expressed in CD4+ T-cells

Etanercept is an anti-TNF drug that can effectively resolve psoriasis, but not all patients show strong improvement while the condition of some may actually worsen [20]. Since we observed differences among patients with respect to inflammatory and cytokine signatures (Figures 6 and 7), we evaluated whether similar metrics could distinguish between etanercept responders and non-responders. For this purpose, we used data from a previous microarray study of biopsies (PP and PN) from patients prior to etanercept treatment, including biopsies from 11 etanercept responders and 4 non-responders [21]. For each of 24 cell types, signatures were calculated based upon the top *N* genes most specifically expressed in that cell type, where the value of *N* was identified by searching for values that maximized separation between responders and non-responders (3 ≤ *N* ≤ 5000; Additional file 11, Part A). Likewise, for each of 42 cytokine experiments, signatures were calculated based upon the top *N* cytokine-induced or cytokine-repressed genes, where the value of *N* was chosen using the same search criteria (3 ≤ *N* ≤ 5000; Additional file 11, Parts B and C).

**Figure 8:**
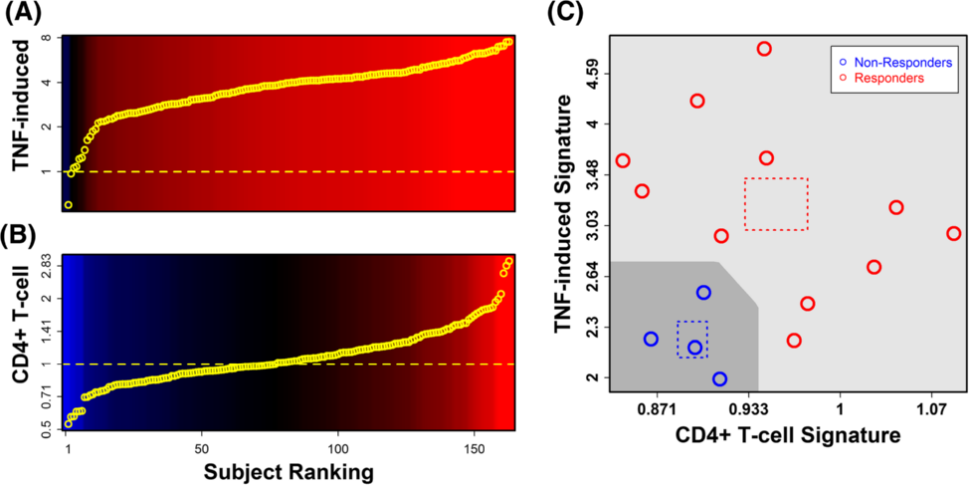
**Signatures calculated from TNF-induced genes and genes specifically expressed in CD4+ T-cells can differentiate between etanercept responders (*n *= 11) and non-responders (*n *= 4).** We identified TNF and CD4+ T-cell signatures that differentiate between etanercept responders and non-responders, based upon paired lesional (PP) and non-lesional (PN) samples obtained at baseline prior to etanercept treatment (Additional file 11). The TNF signature is calculated as the weighted average of fold-changes (PP/PN) of 6 genes induced by treatment of KCs with TNF (10 ng/mL) for 24 hours (GSE36287; Additional file 11, Part B), while the CD4+ T-cell signature is calculated as the weighted average of fold-changes (PP/PN) of 7 genes specifically expressed in CD4+ T-cells (Additional file 11, Part A). Parts **(A)** and **(B)** show the distribution of signature scores among 163 patients. In part **(C)**, signature scores from 11 etanercept responders and 4 non-responders are plotted. Dotted squares outline the bivariate mean for each group (± 1 standard error along each axis). The dark region (lower left) outlines a proposed decision boundary for classification of patients as etanercept non-responders.

Etanercept responders had significantly higher inflammatory signatures, including those calculated for CD3+ T-cells, CD4+ T-cells and B cells (P < 0.05; Additional file 11, Part A). PP skin from responders also had significantly higher expression of genes induced by IL-17C, IL-26d, IL-36b, TGF-α and TNF, with lower expression of genes induced by IL-4 + IL-13 (P < 0.05; Additional file 11, Part B). In addition, the expression of genes repressed by IL-1α, IL-13, IL-36a, IFN-γ and IL-4 + IL-13 was higher in responders, while the expression of genes repressed by IL-17A and IL-17C was lower (P < 0.05; Additional file 11, Part C). Combination of multiple signatures improved differentiation between responders and non-responders. To illustrate, we mapped patients onto the bivariate space formed by TNF-induced and CD4+ T-cell signatures (Figure 8). Among 163 patients, signature scores ranged in value, with some individuals showing no elevation of TNF-induced genes or genes specifically expressed by CD4+ T-cells (Figure 8A and 8B). Only a subset of this variation was observed among the 11 etanercept responders and 4 non-responders (Figure 8C). However, when mapped onto the bivariate signature space (TNF-induced and CD4+ T-cell signature), there was no overlap between responders and non-responders, with clear separation between the centroids calculated for these two groups (dashed boxes, Figure 8C). We could therefore define two distinct regions that completely distinguished the 4 non-responders from the 11 responders (Figure 8C).

## Discussion

Psoriasis lesions arise from complex interactions among infiltrating and resident immune cells, local skin cells, and a network of cytokines, which together create a pro-inflammatory microenvironment that promotes KC proliferation. This working model of psoriasis pathogenesis is consistent with prior microarray studies, all of which have shown increased expression of inflammatory and cytokine-related genes in lesional skin [4-10]. While many differentially expressed genes (DEGs) have been identified within psoriasis lesions, however, underlying mechanisms remain unclear in most cases. In this study, therefore, we robustly identified DEGs based upon a large sample (*n* = 163) and performed targeted analyses to better understand why each DEG is differentially expressed. Our findings show that psoriasis DEGs can be explained in part by shifts in the cellular composition of psoriasis lesions (e.g., KCs and inflammatory cells), and in part by the response of KCs to cytokines, particularly those from the IL-1, IL-17 and IL-20 families. At the same time, among all patients, we uncovered fine-scale differences in the magnitude of inflammatory cell infiltration (56% strong versus 44% weak) and cytokine activity (64% IFN-γ-strong versus 36% IFN-γ-weak). While we could assign mechanisms to explain many psoriasis DEGs, therefore, the relative influence of such mechanisms on gene expression may also vary among lesions. We propose that such inter-patient differences can provide the basis for development of expression-based biomarkers, which might prove useful for predicting individual response to biologic therapies.

Psoriasis lesions and normal human skin consist of a complex mixture of cell types, but key features of lesional skin are an increased presence of inflammatory cells, along with expansion and activation of the resident KC population [22]. For such contexts, we and other investigators have analyzed expression data with the aim of dissecting out the contribution of distinct cell types to observed differences in gene expression between two treatments (e.g., lesional versus non-lesional skin) [6,12,14,15,23-25]. In the present study, our findings indicate that PP-increased DEGs are driven largely by KC activity, including expansion of the KC population in PP skin and the response of KCs to an altered cytokine environment. A fraction of PP-increased DEGs, moreover, could be explained by an influx of inflammatory cells (e.g., γδ T-cells, macrophages and NK cells) (Figures 2 and 4). Interestingly, properties of PP-decreased DEGs contrasted with those of the increased DEGs, since decreased DEGs were commonly expressed by resident cells, including subcutaneous adipose tissue and dermis (Figure 3). We propose that four factors may contribute to this trend. (i) First, in RNA isolates from PP skin, increased proportional contribution of epidermis-derived RNA would necessarily decrease the proportional contribution of other skin cell types. (ii) Second, in one study (GSE13355; 57/163 patients), PN samples were obtained from the buttock/upper thigh region, which tends to have greater subcutaneous adipose tissue and dermal thickness compared to regions where PP samples may often be obtained (e.g., extensor aspects of the extremities) [26-29]. (iii) Third, genes associated with lipid metabolism may be down-regulated in epidermal cells from PP skin [4], and this effect may underlie altered phospholipid abundance in PP skin [30-32]. (iv) Fourth, repressed gene expression may be more easily observed in non-KC skin cells, since abundance of such cell types and their gene products are not, in contrast to KCs, markedly elevated in PP skin. Each of these factors (i) – (iv) would favor decreased expression of adipose- and/or dermis-expressed genes in PP skin (Figure 3). To discriminate among these possibilities, further studies are needed, and along these lines we expect that mRNA profiling of specific cell populations will be most informative (e.g., using flow cytometry or laser capture microdissection) [13].

Psoriasis is understood to be a T-cell-mediated disease and treatments that block interactions between T-cells and antigen presenting cells have demonstrated clinical efficacy [33]. We found that genes specifically expressed in γδ T-cells were better represented among psoriasis-increased DEGs than those genes specifically expressed in other T-cell subsets. This was the case not only in our analysis of bulk skin biopsies (Figure 1), but also of LCM-dissected inflammatory cells from lesional dermis (Additional file 1). This result was obtained largely because genes specifically expressed in γδ T-cells were more *consistently* elevated in psoriasis lesions (Figure 6). We detected significant γδ T-cell signatures in 95% of psoriasis patients, including those from the weak and strong inflammatory groups (Figure 6). In contrast, significant signatures for other T-cell subsets (CD4+, CD8+, natural killer, regulatory) were detected in only 6 – 54% of patients, and most of these patients were from the strong inflammatory group (Figure 6). As a result, genes specifically expressed in other T-cells subsets (CD4+, CD8+, natural killer, regulatory) were not enriched among DEGs, which included only those genes with consistent trends among the 163 patients (Figure 1). In agreement with these findings, previous IHC studies have confirmed increased abundance of γδ T-cells in lesions from psoriasis patients [34-36]. In human skin, absolute numbers of γδ T-cells are modest relative to αβ T-cells [35,37], although this may differ in mouse skin, in which γδ T-cells are estimated to constitute 90% of epidermal T-cells (i.e., many of which are dendritic epidermal T-cells) [38]. Nevertheless, the potential importance of γδ T-cells to psoriasis pathophysiology has been supported by reports demonstrating IL-17A production by γδ T-cells within lesions [34,39,40], by the association between reduced abundance of γδ T-cells in the blood and severity of psoriasis [36], and by the restoration of γδ T-cell numbers in the blood following successful treatment of psoriasis with systemic therapies [36].

Cytokines mediate the inflammatory reactions that sustain KC proliferation, and TNF, IL-12/23, and IL-17A have each been effectively targeted by biologic therapies [41-44]. We compared epidermal PP-increased DEGs to genes induced or repressed by cytokines across a panel of 42 *in vitro* experiments, where each experiment involved treatment of KCs or reconstituted epidermis with a cytokine or cytokine combination. This allowed us to perform an unbiased screen to assess which *in vitro* cytokine expression responses overlapped best with epidermal DEGs, providing indication of which cytokine treatments generate the most “psoriasis-like” expression profile in KCs [16,18]. In some respects, the strongest evidence from our study supports the IL-20 family cytokines as drivers of differential gene expression in psoriasis (i.e., IL-19, IL-20 and IL-24). Genes induced by IL-19, IL-20 and IL-24 overlapped significantly with epidermal genes elevated in PP skin (Figure 4), while conversely, genes repressed by IL-19, IL-20 and IL-24 overlapped significantly with epidermal genes decreased in PP skin (Additional file 7). The transcriptional effects of IL-20 family cytokines, therefore, were associated with both increased and decreased expression in psoriasis lesions. Corresponding trends were observed for each of the three cytokines (IL-19, IL-20 and IL-24), consistent with the observation that each cytokine signals through the same IL-20R1/IL-20R2 receptor complex [45]. Although the importance of IL-20 family cytokines in plaque formation is not completely understood, mRNA and protein levels of IL19, IL20 and IL24 are significantly elevated in psoriatic epidermis [46]. Data from mice suggests that this elevation can augment epidermal hyperplasia, since overexpression of either IL-20 or IL-24 (but not IL-19) elicits a psoriasis-like phenotype [47,48].

Ultimately, events that drive psoriasis plaque formation depend upon activation of a cytokine network, which features interactions among cytokines from multiple families [49]. Along these lines, our findings also support IL-17A and IL-1 (IL-1α/IL-1β) as drivers of increased and decreased DEGs in psoriatic epidermis (Figure 4 and Additional file 7). IL-17A signatures from disparate studies were repeatedly associated with psoriasis DEGs, particularly the PP-increased DEGs (Figure 4). Additionally, of the 42 cytokine treatments screened, genes induced by IL-1α were most strongly enriched among the increased DEGs, while genes repressed by IL-1α were most strongly enriched among decreased DEGs (Figure 4 and Additional file 7). This overlap between IL-1-responsive genes and psoriasis DEGs is consistent with prior work and may reflect the contribution of innate immune responses to the psoriasis transcriptome [16]. Whereas the importance of IL-17A to pathogenesis has now been convincingly demonstrated by successful treatment of patients with IL-17A antibodies [41,50], treatments targeting IL-1 receptor have shown efficacy only for pustular psoriasis, but not plaque psoriasis [51]. Nevertheless, IL-1α potently induces the expression of IL-19, IL-20 and IL-24 in KCs [52,53], drives differentiation of Th17 cells [54], and is required for generation of IL-17A by γδ T-cells in mouse skin [34]. These effects of IL-1β connect together several of the cytokines (and cell types) that were best supported in our study. We therefore speculate that, while the role of IL-1β in psoriasis remains unclear, its activity may nonetheless reinforce activation of the cytokine network within psoriasis lesions.

Cytokines initiate signaling cascades by binding to KC receptors, ultimately leading to the activation or repression of transcription factors (TFs) that coordinate gene expression responses. We screened a dictionary of 1209 TF binding sites to determine which were most strongly enriched in regions 2 KB upstream of genes with significantly altered expression in psoriasis lesions. Among epidermal PP-increased DEGs, we identified nine AP-1 binding sites significantly enriched in 2 KB upstream regions (Figure 5). Additionally, in bulk skin biopsies and in LCM-dissected epidermis, expression of genes encoding AP-1 components was significantly altered (e.g., *JUNB*, *FOSL1* and *FOS*; Additional file 10) [55,56]. These same AP-1 sites, moreover, were similarly enriched in upstream regions of genes induced by IL-17A in KC cultures (also IFN-γ, TNF, IL-20, IL-22 and IL-24; see Figure 5). These results implicate AP-1 as a possible mediator of cytokine-stimulated gene expression in psoriasis lesions, and highlight IL-17A as one potential coordinator of AP-1 activity. In psoriasis lesions, the significance of AP-1 is not yet clear, although AP-1 DNA binding as measured by electrophoretic mobility shift assay is substantially reduced [57]. This attenuation of AP-1 activity could contribute to abnormal KC differentiation, since AP-1 family member genes are differentially expressed in epidermal layers and at different stages of differentiation [58], while expression of a dominant negative form of c-Jun inhibits AP-1 binding in KCs and blocks KC differentiation [59-61]. Furthermore, in mice, double knockout of JunB and c-Jun leads to a skin phenotype with altered KC differentiation, which was suggested to mimic some features of human psoriasis [62,63]. These findings, in combination with our own results, point to AP-1 as a downstream effector of IL-17A (and potentially other cytokines), and support a role for AP-1 in the abnormal KC differentiation characteristically seen in psoriasis lesions.

Psoriasis lesions have characteristic histological features and appear macroscopically similar, but gene expression analyses have also identified sub-groupings among lesions from different individuals [6,7]. Consistent with this, our findings highlight differences in the activation status of inflammatory and cytokine networks in lesions from different patients (Figures 6 and 7). To some degree, we expect that such differences will be associated with early or late stages of plaque development, differences in anatomical location (e.g., trunk versus extremity), or the specific region of a plaque that is sampled (e.g., edge versus center) [6]. On the other hand, the developmental context for each psoriasis lesion is patient-specific and likely shaped by an integration of genetic (e.g., HLA-C genotype, etc.) and environmental signals (e.g., diet, smoking status, sun exposure). Consistent with this idea, we found that inflammatory and cytokine signatures, calculated from baseline biopsies prior to treatment, could differentiate between etanercept responders (*n* = 11) and non-responders (*n* = 4) (Figure 8 and Additional file 11). Etanercept responders, for instance, showed elevated expression of TNF-induced genes as well as genes specifically expressed in CD4+ T-cells (Figure 8). This suggests that variation in gene expression signatures at least partly reflects clinically relevant differences between individuals, potentially due to association with genetic factors that partially determine responses to anti-TNF therapy [64,65]. In future work, therefore, we expect that expression-based signatures, representing inflammatory cell infiltration and cytokine activity, can be integrated with genetic information to create multivariate models that effectively forecast treatment outcomes on an individual basis. This should improve our understanding of factors that underlie treatment responses while also providing a tool that will inform the clinical decision of which type of antipsoriatic therapy should be administered.

## Conclusions

Psoriasis lesions are characterized by large-scale shifts in gene expression, but mechanisms underlying these trends are not completely understood. In this study, we analyzed expression patterns in lesions from a large cohort (*n* = 163 patients) to identify mechanisms driving differentially expressed genes (DEGs). We assigned a candidate cell type for 90% of increased and decreased DEGs, and showed that most increased DEGs can be explained by KC activity and inflammatory cell infiltration (e.g., T-cells and macrophages). Moreover, DEGs expressed highly in epidermis were associated with AP-1 binding sites and were heavily enriched for cytokine-induced genes (e.g., IL-1β, IL-17A and the IL-20 family). We identified sub-groups among the 163 patients based upon signature scores reflecting inflammatory cell infiltration (strong inflammation: 56%; weak inflammation: 44%) and cytokine activity (IFN-γ-strong: 64%; IFN-γ-weak: 36%). Using these signature scores, it was possible to differentiate between etanercept responders (*n* = 11) and non-responders (*n* = 4). Overall, this work advances an analytic framework that can be applied to interpret gene expression in psoriasis or any other inflammatory skin disease. These findings also illustrate the range of gene expression patterns associated with chronic plaque psoriasis, and provide justification for further work exploring the use of expression-based signatures for prediction of treatment outcomes with anti-TNF therapy.

## Methods

### Ethics statement

Procedures were conducted according to the Declaration of Helsinki principles. Informed written consent was obtained from human subjects under protocols approved by the University of Michigan institutional review board (HUM00037994).

### Gene expression datasets

We analyzed paired lesional (PP) and non-lesional (PN) samples from three studies in which gene expression was evaluated using Affymetrix Human Genome Plus 2.0 arrays (GSE13355, GSE14905 and GSE30999) [8,11,19]. In addition to these studies, three other datasets with PP and PN samples have been deposited in Gene Expression Omnibus as of October 2012 (GSE2737, GSE6710 and GSE26866). We did not include these data in our analyses, however, because they were generated using early-generation Affymetrix microarray platforms (Human Genome U95A Array, Human Genome U95 Version 2, Human Genome U133A Array, Human Genome U133A 2.0), with features corresponding to only a limited fraction of known human genes (60% or less). In each of the three included studies (GSE13355, GSE14905 and GSE30999), PP samples were obtained from either the trunk or upper/lower extremities using 4 – 6 mm skin punch biopsies. For at least two of the studies (GSE13355 and GSE30999), PP biopsies were preferentially obtained from the central region of plaques, although in some cases a center could not be clearly discerned due to an irregularly shaped border [8,11]. PN biopsies were always obtained from uninvolved skin with macroscopically normal appearance, but the studies differed with regard to the location of the non-lesional PN biopsy. For GSE14905 and GSE30999, PN biopsies were obtained from an anatomical region similar to that of the PP skin biopsy (e.g., arm, leg or trunk) [8,19]. In contrast, for GSE13355, PN biopsies were always obtained from the sun-protected buttock or upper thigh region, irrespective of the PP biopsy sampling site [11].

### Preprocessing and normalization

CEL files for the three included studies (GSE13355, GSE14905 and GSE30999) were downloaded from Gene Expression Omnibus and Affymetrix quality control (QC) metrics were calculated for each file. These metrics included percentage of probe sets with signals detected above background (percent present), global RNA degradation score, average background, intensity scale factor, and four measures derived from the fitting of probe-level models (RLE median, RLE IQR, NUSE median and NUSE IQR) [66,67]. Samples from each dataset were also clustered to identify potential outliers.

Additional file 12 provides an overview of the preprocessing and filtering procedures. GSE13355 consisted of PP and PN samples from 58 patients with chronic plaque psoriasis [11]. We removed one subject because the PN sample (GSM337287) had high scores on multiple QC metrics (RLE IQR, NUSE median and NUSE IQR). The GSE13355 data had been collected in three separate batches, corresponding to samples processed in 2005, 2006 and 2007, respectively. However, since the paired PP and PN samples for all subjects belonged to the same batch and were processed simultaneously, it was not necessary to adjust for this effect, and we instead normalized data from each batch independently (and calculated PP – PN differences within each batch). GSE14905 consisted of PP and PN samples from 27 patients with chronic plaque psoriasis [19]. We removed one subject because the PP sample (GSM372350) was an outlier in cluster analyses, had a high RNA degradation score, low percentage of probe sets called present, and high scores on other QC metrics (intensity scale factor, RLE median, RLE IQR, NUSE median and NUSE IQR). GSE30999 consisted of PP and PN samples from 81 patients with moderate-to-severe chronic plaque psoriasis [8]. We removed one subject because the PN sample (GSM768062) was an outlier in cluster analyses and had a low percentage of probe sets called present, with high scores on multiple QC metrics (intensity scale factor, RLE median, RLE IQR, NUSE median and NUSE IQR).

After QC filtering, the combined dataset (GSE13355, GSE14905 and GSE30999) included PP and PN samples from 163 patients. Data from GSE14905 and GSE30999 were normalized separately using the robust multi chip average (RMA) method [68]. The three batches from GSE13355 were normalized separately using RMA (see above). The Affymetrix Human Genome Plus 2.0 array includes 54675 probe sets that collectively target 20026 human genes, with the expression of most genes assayed by multiple probe sets [69]. To limit redundancy in our analyses, we *a priori* chose a single probe set to analyze for each of the 20026 human genes. In choosing the representative probe set, we preferentially chose those expected to hybridize specifically with cRNA associated with the targeted gene (i.e., excluding probe sets containing “_x_” or “_s_” in the Affymetrix identifier). If there remained multiple probe sets available for a given gene after applying this criterion, the representative probe set was chosen as the probe set with the highest absolute expression level on average among the 326 PP and PN samples included in our analysis.

### Identification of differentially expressed genes in PP skin versus PN skin

To identify genes differentially expressed between PP and PN samples, the PP – PN difference in RMA expression score was calculated for each probe set and each patient. A linear model was then fit to these differences to identify those probe sets for which the average PP – PN difference was significantly different from zero (*n* = 163). Among the 20026 genes represented on the Human Genome Plus 2.0 array platform, we excluded 1233 that were not significantly expressed above background in any of the 326 PP and PN samples [70]. For the remaining 18793 genes, raw p-values were calculated based upon the empirical Bayes approach and moderated t-statistics implemented in Bioconductor’s limma package [71]. To control the proportion of falsely rejected null hypotheses among all 18793 tests (i.e., false discovery rate), raw p-values were adjusted using the Benjamini-Hochberg method [72]. For analysis of LCM-dissected samples (Figure 4; GSE26866; Affymetrix Human Genome U133A 2.0 array), the same procedures were followed; however, the array platform included probe sets representing only 12711 human genes, of which we excluded 2060 not significantly expressed above background in any of the 37 Affymetrix U133A 2.0 array samples included in our study (i.e., GSE11903 and GSE26866) [13,70].

### Gene set enrichment statistics

In Figures 2A, 2C, 3A, 3C and 4, we report rank-based gene set enrichment statistics. In each case, statistics assess whether genes belonging to a foreground gene set are non-randomly distributed with respect to an independently generated gene ranking [73]. Under the null hypothesis of random association, enrichment statistics are distributed on the [-0.50, 0.50] interval [73]. Positive statistics indicate that genes belonging to the foreground gene set are disproportionately assigned high ranks, while negative statistics indicate that genes belonging to the foreground gene set are disproportionately assigned low ranks. Statistics are proportional to the Wilcoxon Rank Sum test statistic, but can be geometrically interpreted as the difference between two area under the curve (AUC) metrics, based upon “detection rate” curves generated by plotting cumulative percent overlap of a gene set [0,1] relative to gene rank in the reference gene list [73]. In particular, the statistic is calculated as the difference *AUC*_*FG*_ - *AUC*_*BG*_, where *AUC*_*FG*_ is the area under the AUC obtained for the foreground gene set, and *AUC*_*BG*_ is the AUC statistic obtained for the background gene set (usually ≈ 0.50). In our analyses, the foreground gene set is the set of PP-increased (Figures 2 and 4) or PP-decreased genes (Figure 3). The background gene set is the set of all genes measured in both experiments being compared, minus those genes belonging to the foreground gene set. For each statistic, p-values were calculated directly from the standard normal distribution (see equation 8 from Philippakis et al. [73]). For evaluating the overlap between PP-increased or PP-decreased DEGs and cytokine-responsive genes, Fisher’s Exact Test was used as a secondary significance criterion (Figure 4 and Additional file 7). In these cases, significant overlap was indicated by a significantly positive enrichment statistic (FDR < 0.05 by Wilcoxon Rank Sum Test) and significant overlap between DEGs and those genes induced in each cytokine experiment (FDR < 0.05 by Fisher’s Exact Test; cytokine-induced genes defined as those with FC > 1.50 with P < 0.05). Alternatively, significant overlap was indicated by a significantly negative enrichment statistic (FDR < 0.05 by Wilcoxon Rank Sum Test) and significant overlap between DEGs and those genes repressed in each cytokine experiment (FDR < 0.05 by Fisher’s Exact Test; cytokine-repressed genes defined as those with FC < 0.67 with P < 0.05).

### Definition and identification of cell type-specific genes

Among PP-increased and PP-decreased genes, we identified trends related to cell type-specific expression (Figures 1, 2 and 3), and we have calculated signature scores for 163 patients based the expression of “signature genes” specifically expressed in each of 24 cell types (Figures 6 and 7). Throughout the paper, the phrase “cell-type specific” expression is used to denote a gene, or set of genes, for which expression in a given cell type is distinguishably higher than expression in the 23 other cell types evaluated (based upon a two-sample comparison and test for differential expression; see below). This pattern includes genes for which expression can be detected in multiple cell types, provided that expression is higher in one cell type relative to all others. The pattern excludes genes expressed at similarly high levels in multiple cell types, since in such cases a gene’s expression cannot be considered diagnostic of a particular cell type. To identify such genes, we constructed a database with 687 microarray samples, where each sample had been generated using the Affymetrix Human Genome Plus 2.0 array platform.

As an initial step, we searched Gene Expression Omnibus and identified 4145 microarray samples associated with 24 cell types of interest. On average, we identified 173 samples per cell type, with a minimum of 4 (eosinophil and dermis) and a maximum of 799 (CD138+ plasma cell). For 13 of the 24 cell types, more than 40 microarray samples had been identified in our comprehensive screen of Gene Expression Omnibus. In these cases, we identified a minimal set of 40 samples that were most archetypal of all samples identified. To choose these 40 samples, we first used RMA to normalize all samples identified for the cell type. For each sample, we then calculated the average Euclidian distance between that sample and all other samples, which allowed us to identify the 40 samples for which this Euclidean distance was minimized. These 40 samples we chosen as exemplars for the cell type and were thus included in our final database of 687 samples.

After we identified the 687 samples, CEL files for all 687 samples were jointly normalized using RMA. As noted above, the Affymetrix Human Genome Plus 2.0 array includes 54675 probe sets collectively targeting 20026 human genes. In our analyses, we chose a single representative probe set for each human gene, and for consistency, we used the same probe sets selected based upon the criteria stated above. To identify genes with cell type-specific expression, a two-sample comparison was made between those samples associated with a given cell type and all other samples in the database. For each cell type, this comparison was performed for each gene and p-values were generated using the empirical Bayes approach and moderated t-statistics implemented in Bioconductor’s limma package [71]. In Figure 6, this procedure was used to identify the 250 signature genes, by selecting those genes with lowest p-values and for which expression was increased in the cell type of interest relative to all other cell types (FC > 1; also see section below on signature score calculation). Likewise, in Figures 2A, 2C, 3A and 3C, this procedure was followed to generate the p-values used to rank all genes according to evidence for cell type-specific expression. Genes with FC > 1 were ranked ascendingly by p-values, followed by genes with FC < 1, which were ranked descendingly by p-values. After ranking genes in this fashion, we evaluated whether a set of DEGs (PP-increased or PP-decreased) was disproportionately assigned higher ranks using enrichment statistics and the Wilcoxon rank sum test [73] (see above section on gene set enrichment statistics).

### Identification of cytokine-responsive genes

Cytokine-responsive genes were identified based upon 42 experiments in which cultured KCs or reconstituted epidermis had been treated with cytokines (Additional file 3). Raw data from each experiment is available from Gene Expression Omnibus (GSE9120, GSE25400, GSE2737, GSE36287, GSE12109, GSE24767, GSE32620, GSE1132, GSE440, GSE8531, GSE7661, GSE2489, GSE36387, GSE17892, GSE32975, GSE20706 and GSE20297). For experiments that evaluated expression using Affymetrix platforms, raw data were downloaded and normalized using RMA. For all other platforms, we used normalized data available in GEO series matrix files. Differential expression between cytokine-treated and untreated control cells was evaluated using the empirical Bayes approach and moderated t-statistics as implemented in Bioconductor’s limma package [71]. For most array platforms, multiple probes were available to measure expression for a given human gene. To limit redundancy, only a single probe was analyzed as a representative of each human gene. For each experiment, this representative probe was chosen as the one with the lowest p-value from differential expression analysis comparing cytokine-treated cells with untreated control cells.

### Calculation of signature scores

Signature scores were calculated as the weighted average of fold-changes (PP/PN) among cytokine-responsive genes (Figure 7) or genes specifically expressed in a given cell type (Figure 6). For each signature, a set of *N* ranked genes was defined and signature scores were calculated using the weighted arithmetic mean of PP/PN fold-changes (R function “weighted.mean”). Calculations were performed using log_2_-transformed fold-changes, although in our results we have reported signature scores on an untransformed scale (Figures 6, 7 and 8). The weight of each gene was equal to the square root of its rank, with the top-ranked gene assigned a weight of *N*^1/2^, the next-ranked gene assigned a weight of (*N*-1)^1/2^, and so on, with the last-ranked gene assigned a weight of 1^1/2^. In Figure 6, the top-ranked signature genes were those with the highest fold-change expression difference (*x*/*y*), where *x* is the average expression in a given cell type, and *y* is the average expression of the gene in the other 23 cell types evaluated (see Figure 1). In Figure 7, top-ranked signature genes were those with the highest fold-change induction following cytokine treatment (treated/control; see Additional files 4 and 6). In all cases, the *N* signature genes were identified by first selecting the 1.5*N* genes with lowest p-value, where p-values were generated from differential expression analyses identifying genes with cell type-specific expression (Figure 6) or genes induced by cytokine treatment (Figure 7). These 1.5*N* genes were then sorted descendingly according to fold-change, and the top *N* genes with highest fold-change were selected as the signature genes. In Additional file 11, the same procedures were followed to identify *N* signature genes, except gene weights were assigned based upon rankings determined either by p-values or fold-change estimates, depending upon which approach led to better separation between etanercept responders and non-responders.

### Motif analyses

We screened 1209 transcription factor (TF) binding site motifs to evaluate whether such motifs are enriched among epidermal PP-increased DEGs (Figure 5). These 1209 non-redundant motifs were derived from the Jaspar [74], UniPROBE [75] and TRANSFAC [76] databases, as described in a recent research report [77]. Regions 2KB upstream of human genes were scanned for matches to the 1209 binding sites. Sequences were obtained from Bioconductor (BSgenome.Hsapiens.UCSC.hg19) with coordinates for each gene defined based upon UCSC refGene files (hg19). Assembly gaps, repetitive DNA and coding regions were masked for all genome scans. Motif matches were identified based upon position weight matrices (PWMs), with a match identified only for those loci for which the PWM matching score was greater than 80% of the maximum matching score for that PWM matrix [15,77,78].

We defined foreground (FG) and background (BG) gene sets and tested whether any motifs were differentially abundant in the regions upstream of genes belonging to each set. In Figure 5, the FG set included 709 PP-increased genes specifically expressed in KCs or epidermis, and the BG set included the 18084 other genes included in our analysis (Affymetrix Human Genome Plus 2.0 array). In Additional file 9, the FG gene set included 900 genes elevated in LCM-dissected epidermis from lesional skin relative to LCM-dissected epidermis from normal skin, and the BG set included the 9751 other genes included in our analysis and represented on the array platform (Affymetrix Human Genome U133A 2.0 array). A semiparametric generalized additive logistic model (GAM) was used to test whether abundance of each motif differed between genes in the FG and BG sets [77,79]. In GAM models, the response variable was an indicator with value equal to 1 if a gene belonged to the FG set and a value of 0 if a gene belonged to the BG set. Each GAM model included two predictor variables, including log-transformed length of upstream sequence scanned for a given gene (*x*_1_; non-parametric term with cubic spline smoothing) and the number of motif sites detected within the upstream sequence (*x*_2_; parametric term without smoothing) [77]. The association between motif frequency and gene set membership was evaluated based upon the coefficient estimate associated with *x*_2_. Separate models were fit for all 1209 motifs and p-values were calculated for each motif by comparing Z statistics to the standard normal distribution [77]. Raw p-values were adjusted to control the false discovery rate by applying the Benjamini-Hochberg correction [72].

## Competing interests

The authors declare that they have no competing interests.

## Authors’ contributions

WRS, AJ and JEG participated in the design of the study, analysis of data and drafting the manuscript. JJV and JTE assisted in drafting the manuscript and revising it critically. All authors have read and approved of the final manuscript.

## Supplementary Material

Additional file 1 **Genes significantly elevated in dermal inflammatory cells from PP skin are specifically expressed in NK cells, macrophages, dendritic cells, B cells, monocytes and multiple T-cell subsets.** Gene expression was compared between LCM-dissected dermal inflammatory cells from PP skin and LCM-dissected dermis from uninvolved skin (*n* = 3 patients; data from GEO accession GSE26866). Based on this comparison, we identified 609 genes with significantly elevated expression in dermal inflammatory cells from PP skin (P < 0.05 and FC > 1.50). The analysis shown in Figure 2 was performed starting with these 609 genes. Click here for file

Additional file 2 **Cell type-specific expression of the 35 genes most strongly decreased in psoriasis lesions (PP) relative to uninvolved skin (PN).** The left margin lists the 35 genes most strongly decreased in PP skin relative to PN skin (FDR < 0.05; ranked according to PP/PN fold-change). Heatmap colors show fold-change estimates for each of 24 cell types (columns), with fold-changes estimated as the ratio of a gene’s expression in a given cell type (numerator), relative to its expression among the 23 other cell types (denominator). Triangle symbols denote cases in which gene expression is significantly altered in one cell type as compared to all other cell types (see legend). Click here for file

Additional file 3 **List of 42 cytokine experiments (primary monolayer KC cultures, HaCaT KCs or 3-D reconstituted epidermis).** The table lists experiments in which gene expression responses were evaluated in cytokine-treated cells. The label for each experiment indicates the cytokine used, the concentration (per mL), the length of time cells were treated, and the Gene Expression Omnibus accession under which raw data can be accessed. The third column lists the microarray platform used to evaluate gene expression in each experiment and the corresponding Gene Expression Omnibus platform identification number. Further details on each experiment are available from Gene Expression Omnibus or the reference listed in the final column. Click here for file

Additional file 4 **Cytokine responses of the 30 epidermal genes most strongly increased in psoriasis lesions (PP) relative to uninvolved skin (PN).** The left margin lists the 30 epidermal genes most strongly increased in PP skin relative to PN skin (FDR < 0.05; ranked according to PP/PN fold-change). Heatmap colors show the expression response of each gene across 42 cytokine experiments (top margin). In each experiment, KCs or 3-D reconstituted epidermis was treated with cytokines and microarrays were used to measure changes in gene expression. Experiments using HaCaT KCs are indicated with a single asterisk symbol (*), while experiments using reconstituted epidermis are indicated by a double asterisk (**). All other experiments utilized primary monolayer KC cultures. Labels list the cytokine used, the concentration (per mL), the length of time cells were treated, and the Gene Expression Omnibus accession under which raw data can be accessed. Click here for file

Additional file 5 **80% of epidermal PP-increased DEGs can be explained as gene expression responses of KCs to cytokine stimulation.** We identified 709 epidermal PP-increased genes (Figure 2) and showed that these genes were disproportionately induced or repressed in 35 experiments in which KCs (or reconstituted epidermis) had been treated with cytokines (Figure 4). We assigned each DEG to one of these 35 experiments, depending upon whether the DEG was significantly induced or repressed (P < 0.05; also FC > 1.5 for experiments with red labels, or FC < 0.67 for experiments with blue labels). DEGs were preferentially assigned to the experiment for which induced or repressed genes overlapped most significantly with the complete set of 709 epidermal PP-increased genes (Wilcoxon Rank Sum Test; Figure 4). The chart shows the number of DEGs assigned to each experiment, where the “non-responsive” category includes those DEGs not significantly altered in any of the 35 experiments. In the left margin, red labels denote those experiments for which induced genes overlapped significantly with the 709 epidermal PP-increased DEGs, while blue labels denote those experiments for which repressed genes overlapped significantly with the 709 DEGs (see Figure 4). Click here for file

Additional file 6 **Cytokine responses of the 30 epidermal genes most strongly decreased in psoriasis lesions (PP) relative to uninvolved skin (PN).** The left margin lists the 30 epidermal genes most strongly decreased in PP skin relative to PN skin (FDR < 0.05; ranked according to PP/PN fold-change). Heatmap colors show the expression response of each gene across 42 cytokine experiments (top margin). In each experiment, KCs or 3-D reconstituted epidermis was treated with cytokines and microarrays were used to measure changes in gene expression. Experiments using HaCaT KCs are indicated with a single asterisk symbol (*), while experiments using 3-D reconstituted epidermis are indicated by a double asterisk (**). All other experiments utilized primary monolayer KC cultures. Labels list the cytokine used, the concentration (per mL), the length of time cells were treated, and the Gene Expression Omnibus accession under which raw data can be accessed. Click here for file

Additional file 7 **Epidermal genes decreased in psoriasis lesions overlap best with genes repressed by IL-10/IL-20 family cytokines in cultured KCs.** The analysis shown in Figure 4 was repeated based upon (A) 134 epidermal PP-decreased DEGs (FDR < 0.05 & FC > 1.50) and (B) 876 genes decreased in LCM-dissected PP epidermis relative to LCM-dissected epidermis from uninvolved skin (FDR < 0.05 & FC < 0.67). Click here for file

Additional file 8 **60% of epidermal PP-decreased DEGs can be explained as gene expression responses of KCs to cytokine stimulation.** We identified 135 epidermal PP-decreased genes (Figure 3) and showed that these genes were disproportionately repressed in 10 experiments in which KCs (or reconstituted epidermis) had been treated with cytokines (Additional file 7). We assigned each DEG to one of these 10 experiments, depending upon whether the DEG was significantly repressed (P < 0.05 and FC < 0.67). DEGs were preferentially assigned to the experiment for which induced or repressed genes overlapped most significantly with the complete set of 135 epidermal PP-decreased genes (Wilcoxon Rank Sum Test; Additional file 7). The chart shows the number of DEGs assigned to each experiment, where the “non-responsive” category includes those DEGs not significantly repressed in any of the 10 experiments. For two experiments (IL-20/GSE7216 and TNF/GSE2489), none of the DEGs met our assignment criteria and thus only 8 of the 10 experiments are shown in the Figure. Click here for file

Additional file 9 **Transcription factor binding sites most enriched in 2KB regions upstream of 900 genes elevated in LCM-dissected PP epidermis from psoriasis lesions.** The analysis shown in Figure 5 was repeated starting with 900 genes elevated in LCM-dissected epidermis from psoriasis lesions (compared to LCM-dissected epidermis from normal skin; FDR < 0.05 and FC > 1.50). The left margin lists the top 25 motifs most strongly enriched in 2KB regions upstream of the 900 genes (P ≤ 0.014 and FDR ≤ 0.49). Values in the bottom margin list statistics calculated for each cytokine experiment, which assess whether the 900 genes are disproportionately elevated or repressed in a given cytokine experiment (see Figure 4B). Magenta labels denote motifs recognized by the AP-1 complex. Click here for file

Additional file 10 **Genes encoding components of the AP-1 complex are differentially expressed between lesional (PP) and uninvolved skin (PN).** (A) Fold-changes (PP/PN) for genes encoding components of the AP-1 complex were evaluated in 163 patients. Grey boxes outline the middle 50% of fold-change estimates for each gene. (B) Expression of AP-1 component genes was evaluated in LCM-dissected epidermis from PP skin and LCM-dissected epidermis from PN skin (*n* = 3). (C) Expression of AP-1 component genes was evaluated in LCM-dissected dermis from PP skin and LCM-dissected dermis from PN skin (*n* = 3). In (B) and (C), asterisk symbols denote genes with significantly altered expression (P < 0.05). In (A) – (C), red labels denote genes with significantly elevated expression in lesional skin, while blue labels denote genes with significantly decreased expression in lesional skin (P < 0.05). Click here for file

Additional file 11 **Identification of inflammatory and cytokine signatures that distinguish etanercept responders from non-responders.** (A) For each cell population, signature scores were calculated as the weighted average of fold-changes (PP/PN) among the top *N* cell type-specific genes (weighted arithmetic mean). The value of *N* is listed in the top margin for each cell type, and was chosen by searching for values (3 ≤ *N* ≤ 5000) that maximized separation between responders and non-responders (i.e., minimized the p-value obtained from a two-sample *t*-test). In signature calculations, genes were weighted according to the square root of their rank (see Methods). Genes were ranked either by p-values generated from the test for cell type-specific expression (asterisk; top margin), or were ranked by the fold-change ratio of a gene’s expression in a given cell type relative to the 23 other cell types (no asterisk; top margin). The ranking approach used for a given threshold was the one leading to better separation between responders and non-responders. Red labels indicate cell populations for which signature scores of responders were at least marginally higher than those of non-responders (P < 0.10; two-sample *t*-test). Conversely, blue labels denote cell types for which signature scores of non-responders were at least marginally higher than those of responders (P < 0.10; two-sample *t*-test). Italicized labels denote cases in which signature scores for responders and non-responders differed significantly (P < 0.05). In parts (B) and (C), the same analyses were performed, except signature scores were calculated based upon (B) the *N* genes most strongly induced in each of 42 cytokine experiments or (C) the *N* genes most strongly repressed in each of 42 cytokine experiments. Click here for file

Additional file 12 **Overview of data processing steps leading to detection of differentially expressed genes.** Paired lesional (PP) and uninvolved (PN) microarray samples generated using the same platform (Affymetrix Human Genome Plus 2.0 array) were obtained from each of three studies (GSE13355, GSE14905 and GSE30999). Following quality control (QC) filtering, CEL files from each study were normalized using robust multichip average (RMA). Samples from GSE13355 were collected in three batches and each batch was normalized separately. Paired PP – PN differences were next calculated for all genes within each dataset, and these differences were subsequently pooled. For each gene, this yielded PP – PN expression differences from 163 patients. We removed from consideration 1233 genes not significantly expressed above background in any of the PP and PN samples. For the remaining 18793 genes, we tested whether the mean PP – PN expression difference (log2 scale) was significantly different from zero (moderated *t*-test). This led to the identification of 1233 PP-increased DEGs (FDR < 0.05 and FC > 1.50) and 977 PP-decreased DEGs (FDR < 0.05 and FC < 0.67). Click here for file
