# Supplementary figures and images for: Dissecting the psoriasis transcriptome: inflammatory- and cytokine-driven gene expression in lesions from 163 patients
Source: BMC Genomics. 2013 Aug 1;14:527. doi: 10.1186/1471-2164-14-527 (PMC3751090; doi:10.1186/1471-2164-14-527)

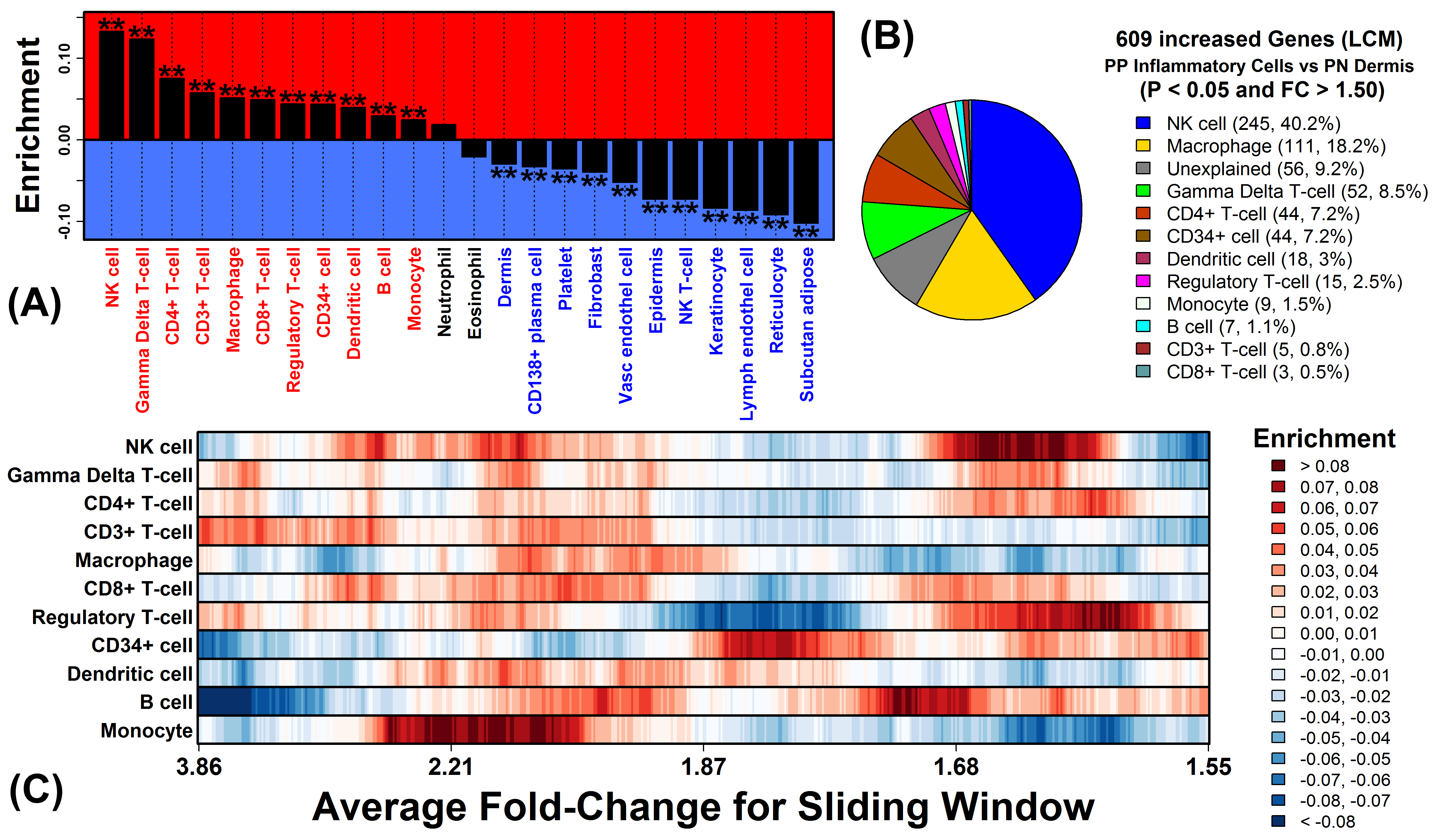

Supplement: Additional file 1 — Genes significantly elevated in dermal inflammatory cells from PP skin are specifically expressed in NK cells, macrophages, dendritic cells, B cells, monocytes and multiple T-cell subsets. Gene expression was compared between LCM-dissected dermal inflammatory cells from PP skin and LCM-dissected dermis from uninvolved skin (n = 3 patients; data from GEO accession GSE26866). Based on this comparison, we identified 609 genes with significantly elevated expression in dermal inflammatory cells from PP skin (P < 0.05 and FC > 1.50). The analysis shown in Figure 2 was performed starting with these 609 genes. [file 1471-2164-14-527-S1.tiff]

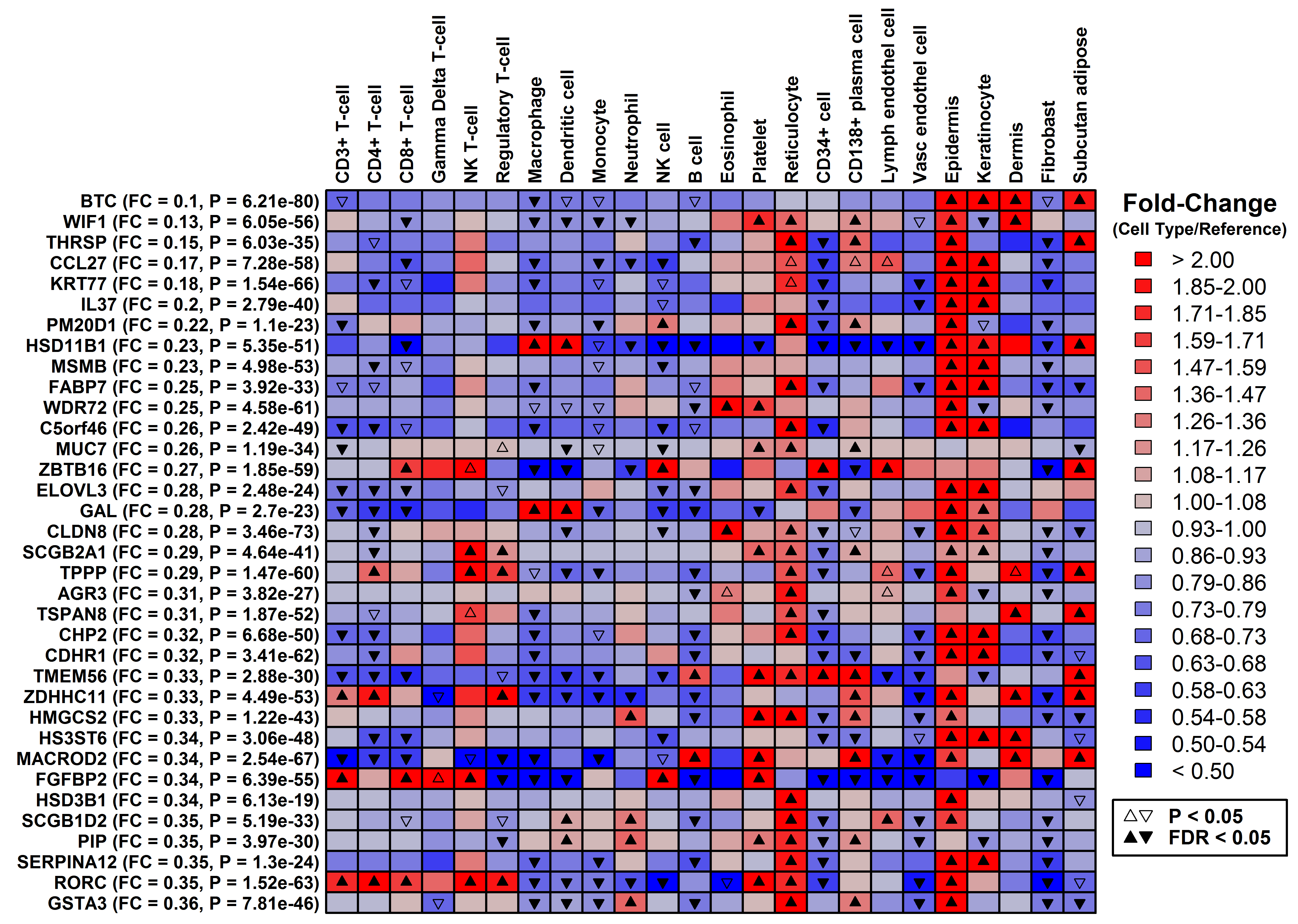

Supplement: Additional file 2 — Cell type-specific expression of the 35 genes most strongly decreased in psoriasis lesions (PP) relative to uninvolved skin (PN). The left margin lists the 35 genes most strongly decreased in PP skin relative to PN skin (FDR < 0.05; ranked according to PP/PN fold-change). Heatmap colors show fold-change estimates for each of 24 cell types (columns), with fold-changes estimated as the ratio of a gene’s expression in a given cell type (numerator), relative to its expression among the 23 other cell types (denominator). Triangle symbols denote cases in which gene expression is significantly altered in one cell type as compared to all other cell types (see legend). [file 1471-2164-14-527-S2.tiff]

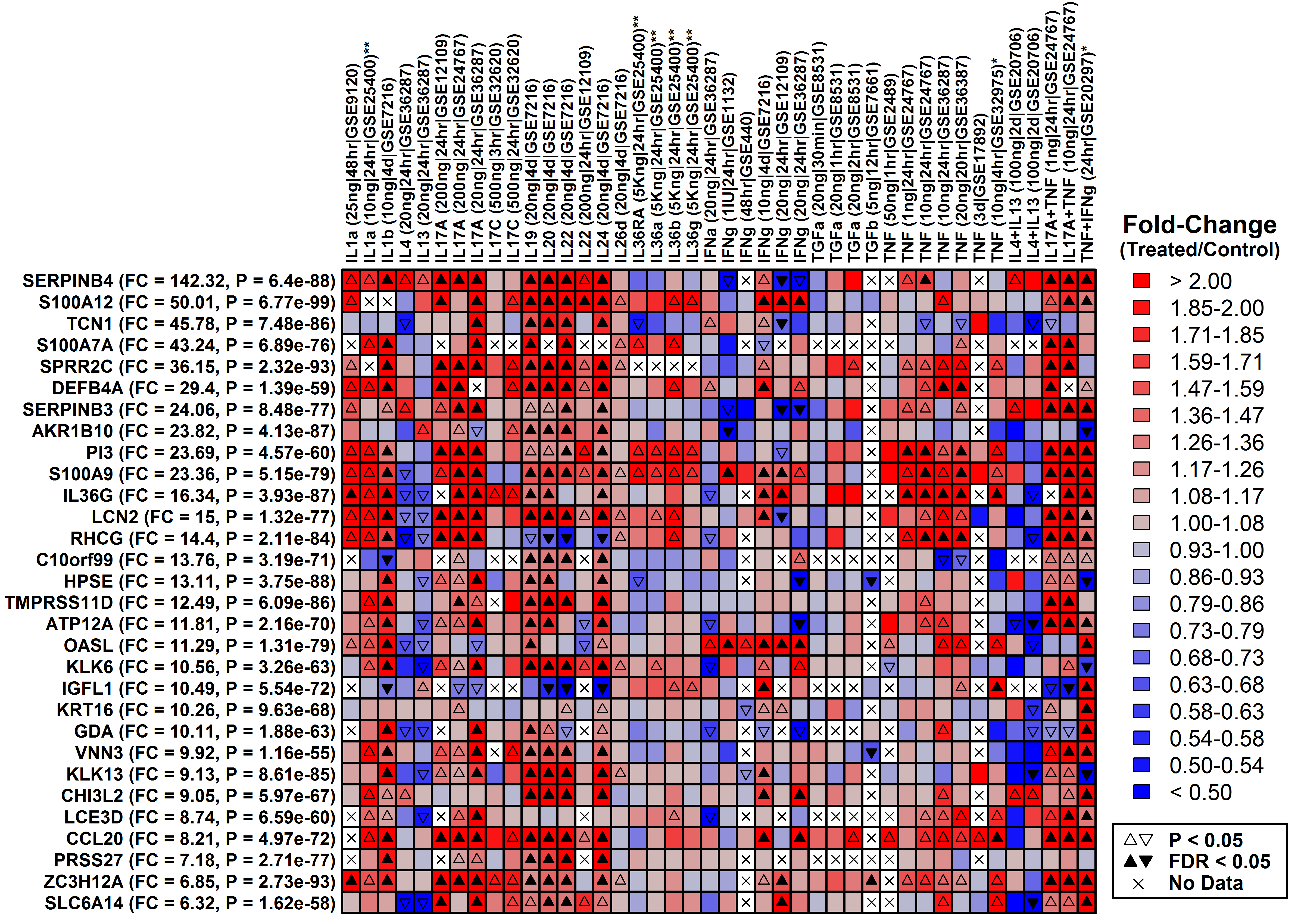

Supplement: Additional file 4 — Cytokine responses of the 30 epidermal genes most strongly increased in psoriasis lesions (PP) relative to uninvolved skin (PN). The left margin lists the 30 epidermal genes most strongly increased in PP skin relative to PN skin (FDR < 0.05; ranked according to PP/PN fold-change). Heatmap colors show the expression response of each gene across 42 cytokine experiments (top margin). In each experiment, KCs or 3-D reconstituted epidermis was treated with cytokines and microarrays were used to measure changes in gene expression. Experiments using HaCaT KCs are indicated with a single asterisk symbol (*), while experiments using reconstituted epidermis are indicated by a double asterisk (**). All other experiments utilized primary monolayer KC cultures. Labels list the cytokine used, the concentration (per mL), the length of time cells were treated, and the Gene Expression Omnibus accession under which raw data can be accessed. [file 1471-2164-14-527-S4.tiff]

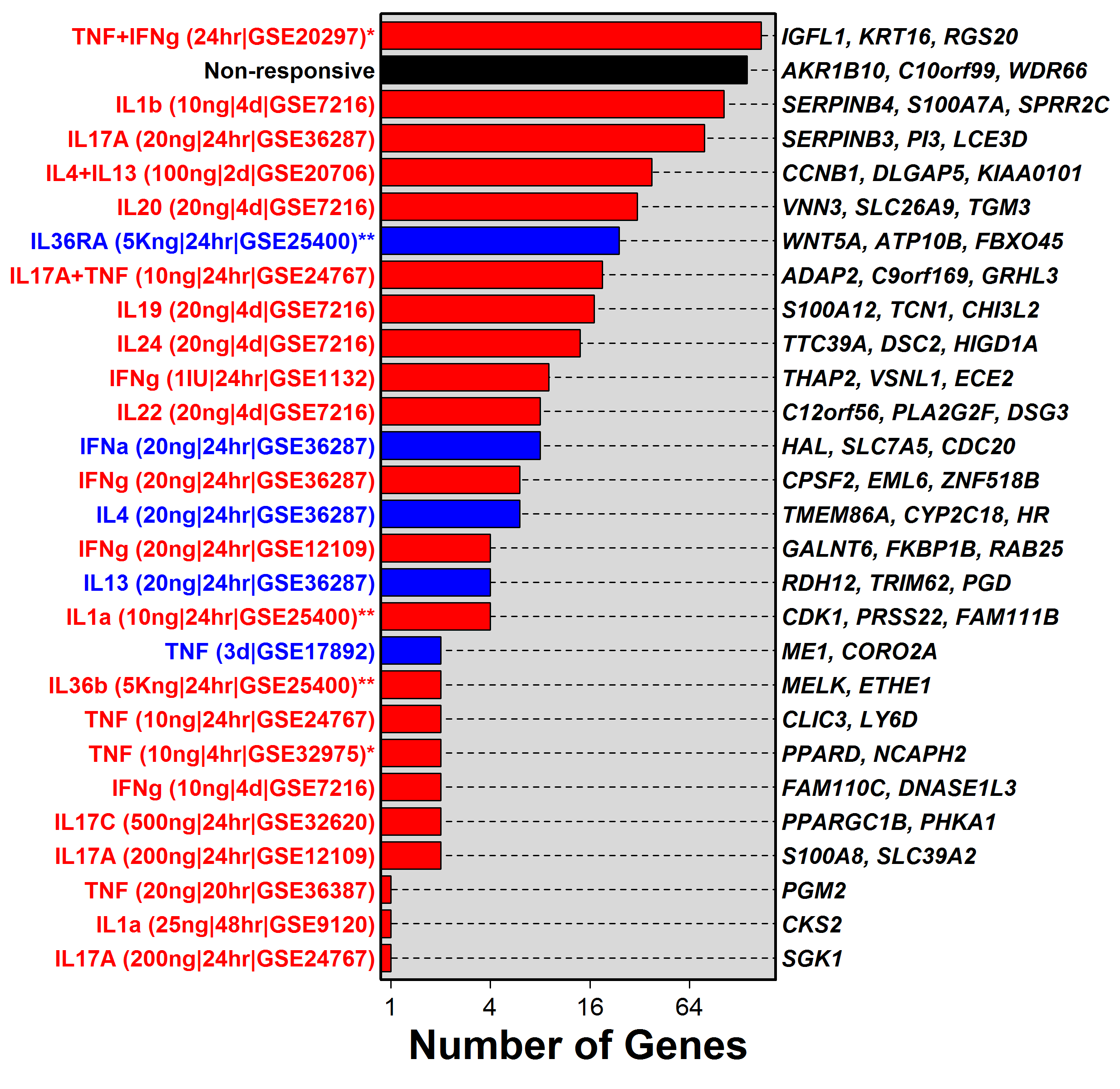

Supplement: Additional file 5 — 80% of epidermal PP-increased DEGs can be explained as gene expression responses of KCs to cytokine stimulation. We identified 709 epidermal PP-increased genes (Figure 2) and showed that these genes were disproportionately induced or repressed in 35 experiments in which KCs (or reconstituted epidermis) had been treated with cytokines (Figure 4). We assigned each DEG to one of these 35 experiments, depending upon whether the DEG was significantly induced or repressed (P < 0.05; also FC > 1.5 for experiments with red labels, or FC < 0.67 for experiments with blue labels). DEGs were preferentially assigned to the experiment for which induced or repressed genes overlapped most significantly with the complete set of 709 epidermal PP-increased genes (Wilcoxon Rank Sum Test; Figure 4). The chart shows the number of DEGs assigned to each experiment, where the “non-responsive” category includes those DEGs not significantly altered in any of the 35 experiments. In the left margin, red labels denote those experiments for which induced genes overlapped significantly with the 709 epidermal PP-increased DEGs, while blue labels denote those experiments for which repressed genes overlapped significantly with the 709 DEGs (see Figure 4). [file 1471-2164-14-527-S5.tiff]

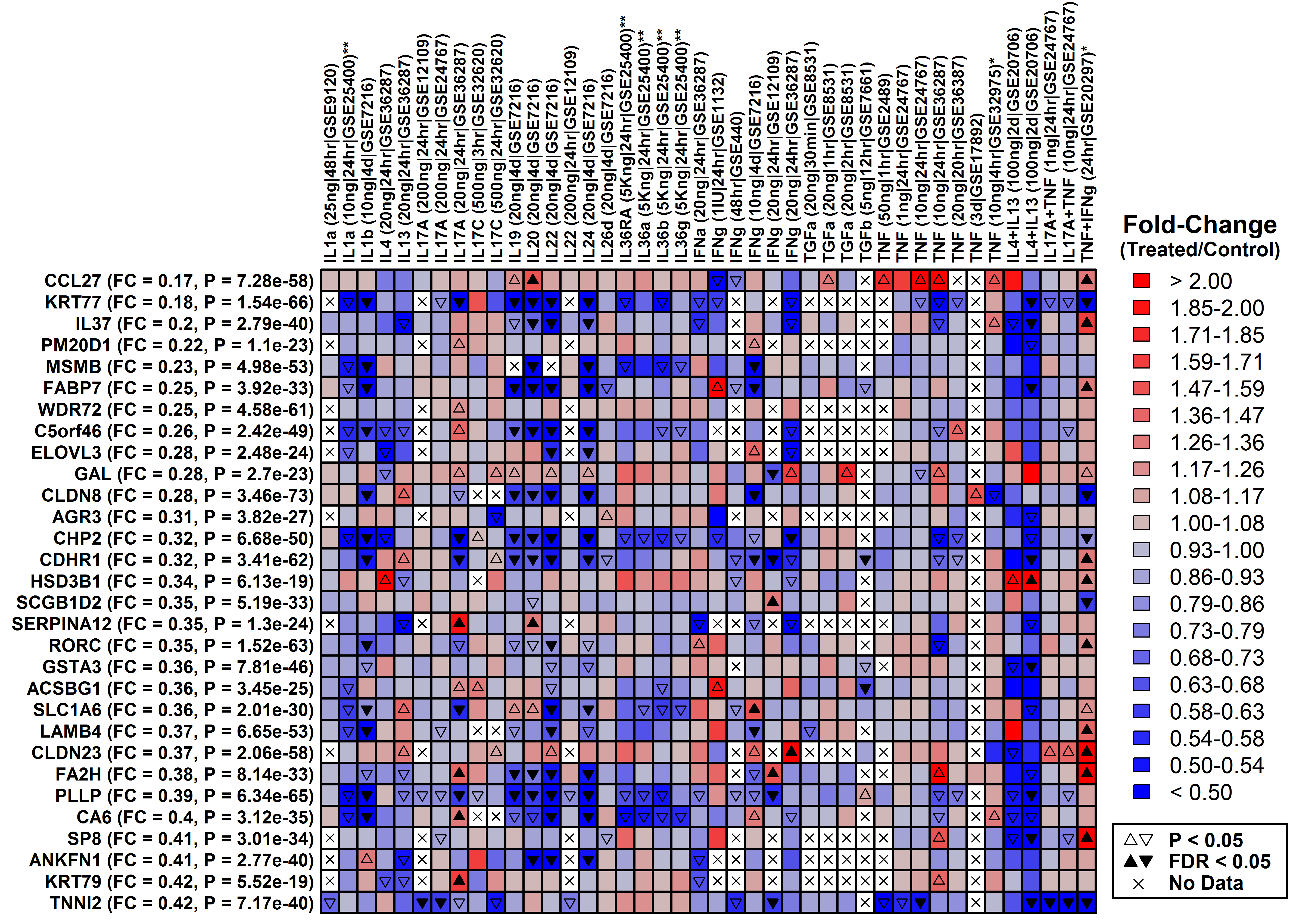

Supplement: Additional file 6 — Cytokine responses of the 30 epidermal genes most strongly decreased in psoriasis lesions (PP) relative to uninvolved skin (PN). The left margin lists the 30 epidermal genes most strongly decreased in PP skin relative to PN skin (FDR < 0.05; ranked according to PP/PN fold-change). Heatmap colors show the expression response of each gene across 42 cytokine experiments (top margin). In each experiment, KCs or 3-D reconstituted epidermis was treated with cytokines and microarrays were used to measure changes in gene expression. Experiments using HaCaT KCs are indicated with a single asterisk symbol (*), while experiments using 3-D reconstituted epidermis are indicated by a double asterisk (**). All other experiments utilized primary monolayer KC cultures. Labels list the cytokine used, the concentration (per mL), the length of time cells were treated, and the Gene Expression Omnibus accession under which raw data can be accessed. [file 1471-2164-14-527-S6.tiff]

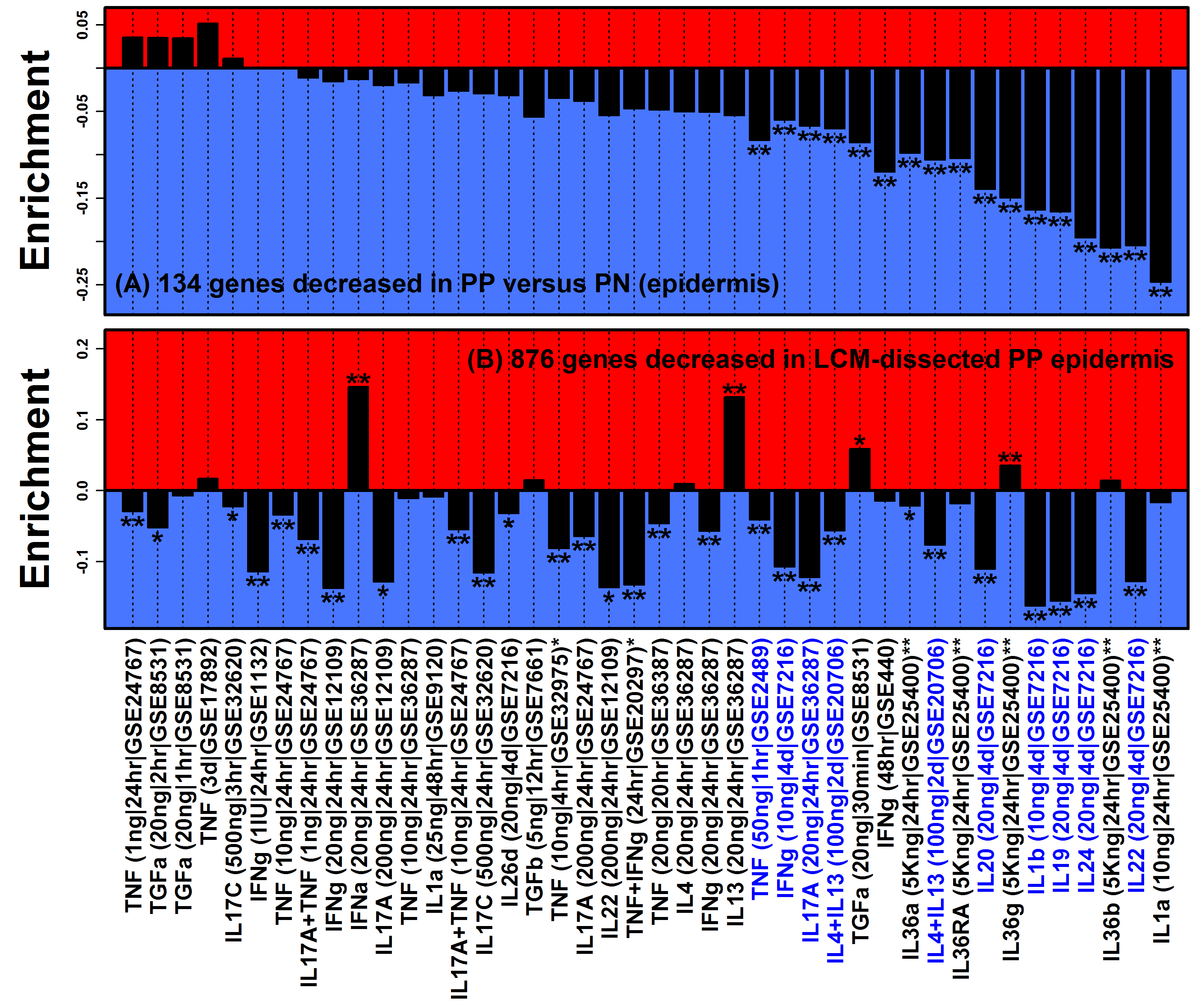

Supplement: Additional file 7 — Epidermal genes decreased in psoriasis lesions overlap best with genes repressed by IL-10/IL-20 family cytokines in cultured KCs. The analysis shown in Figure 4 was repeated based upon (A) 134 epidermal PP-decreased DEGs (FDR < 0.05 & FC > 1.50) and (B) 876 genes decreased in LCM-dissected PP epidermis relative to LCM-dissected epidermis from uninvolved skin (FDR < 0.05 & FC < 0.67). [file 1471-2164-14-527-S7.tiff]

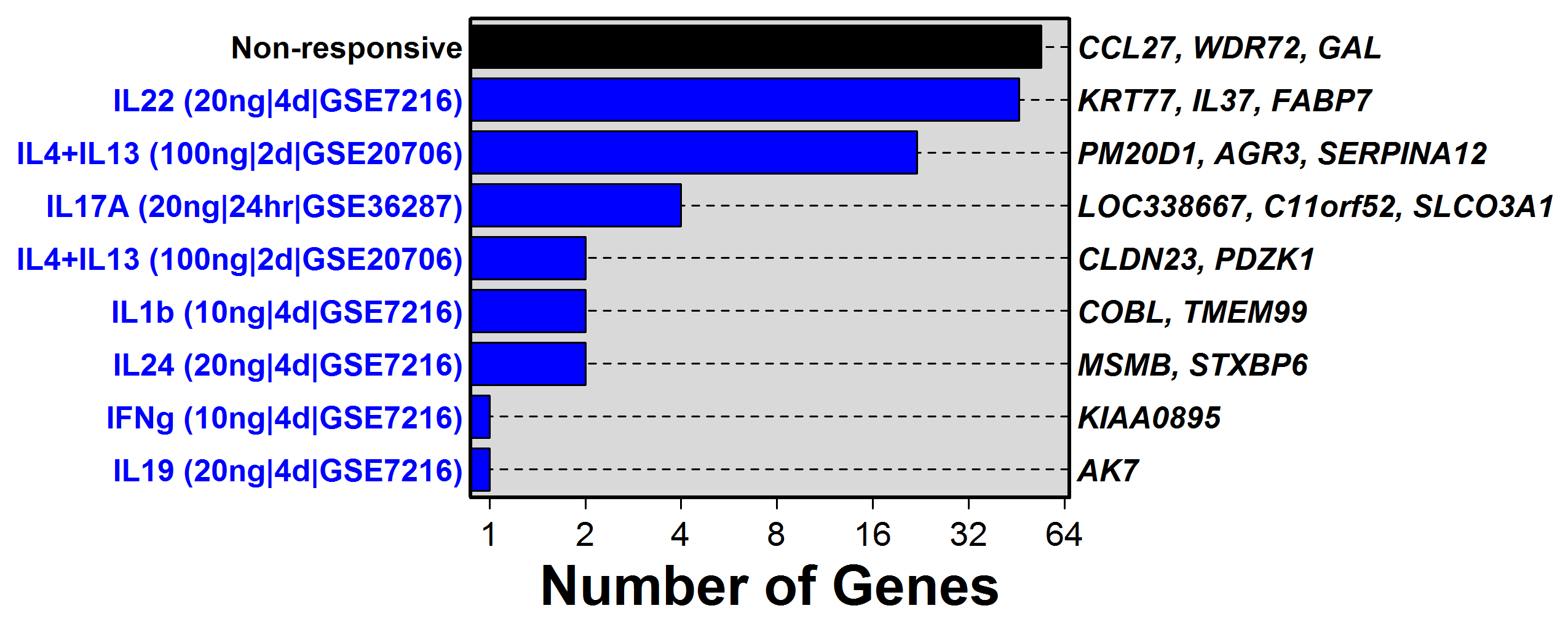

Supplement: Additional file 8 — 60% of epidermal PP-decreased DEGs can be explained as gene expression responses of KCs to cytokine stimulation. We identified 135 epidermal PP-decreased genes (Figure 3) and showed that these genes were disproportionately repressed in 10 experiments in which KCs (or reconstituted epidermis) had been treated with cytokines (Additional file 7). We assigned each DEG to one of these 10 experiments, depending upon whether the DEG was significantly repressed (P < 0.05 and FC < 0.67). DEGs were preferentially assigned to the experiment for which induced or repressed genes overlapped most significantly with the complete set of 135 epidermal PP-decreased genes (Wilcoxon Rank Sum Test; Additional file 7). The chart shows the number of DEGs assigned to each experiment, where the “non-responsive” category includes those DEGs not significantly repressed in any of the 10 experiments. For two experiments (IL-20/GSE7216 and TNF/GSE2489), none of the DEGs met our assignment criteria and thus only 8 of the 10 experiments are shown in the Figure. [file 1471-2164-14-527-S8.tiff]

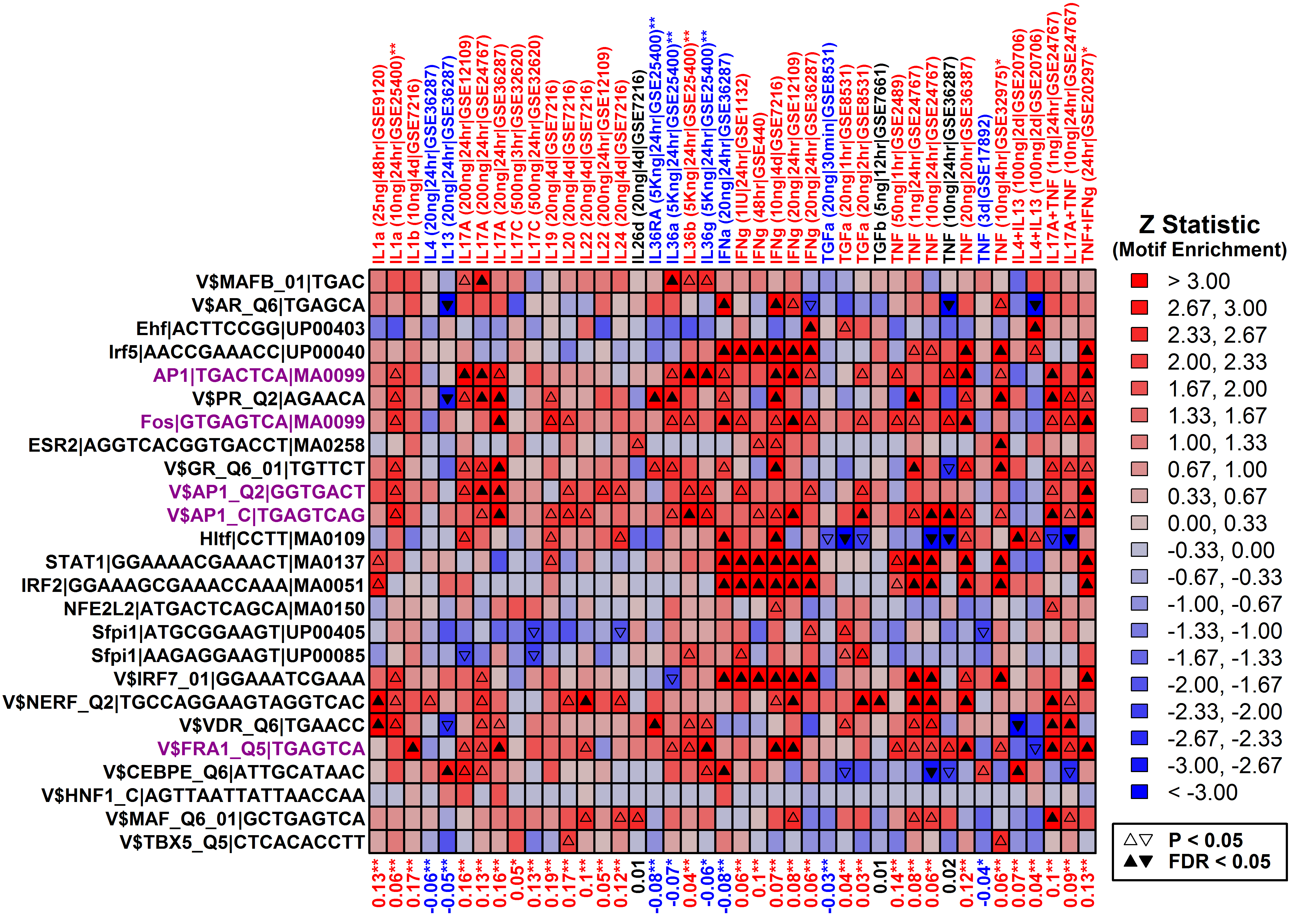

Supplement: Additional file 9 — Transcription factor binding sites most enriched in 2KB regions upstream of 900 genes elevated in LCM-dissected PP epidermis from psoriasis lesions. The analysis shown in Figure 5 was repeated starting with 900 genes elevated in LCM-dissected epidermis from psoriasis lesions (compared to LCM-dissected epidermis from normal skin; FDR < 0.05 and FC > 1.50). The left margin lists the top 25 motifs most strongly enriched in 2KB regions upstream of the 900 genes (P ≤ 0.014 and FDR ≤ 0.49). Values in the bottom margin list statistics calculated for each cytokine experiment, which assess whether the 900 genes are disproportionately elevated or repressed in a given cytokine experiment (see Figure 4B). Magenta labels denote motifs recognized by the AP-1 complex. [file 1471-2164-14-527-S9.tiff]

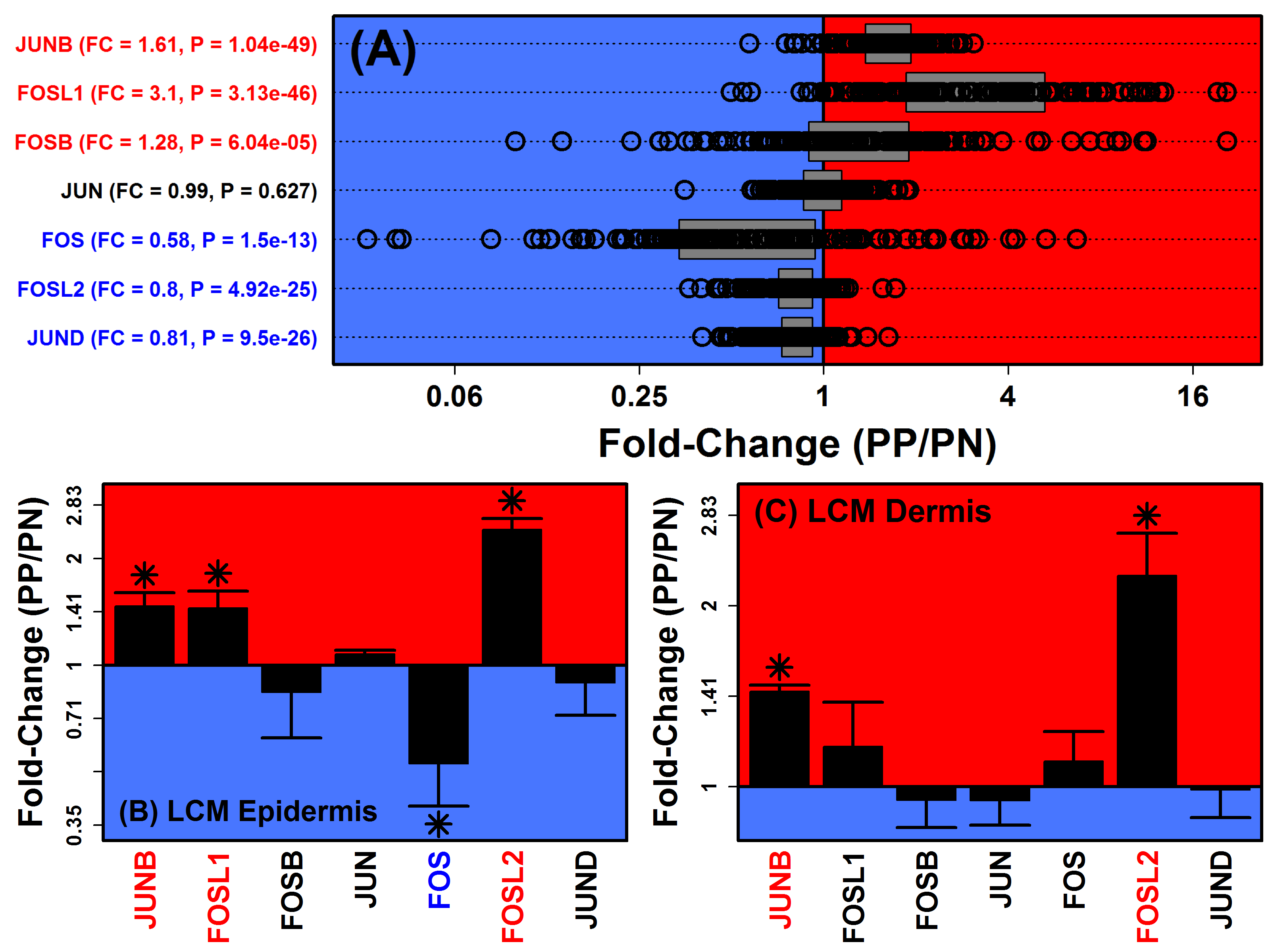

Supplement: Additional file 10 — Genes encoding components of the AP-1 complex are differentially expressed between lesional (PP) and uninvolved skin (PN). (A) Fold-changes (PP/PN) for genes encoding components of the AP-1 complex were evaluated in 163 patients. Grey boxes outline the middle 50% of fold-change estimates for each gene. (B) Expression of AP-1 component genes was evaluated in LCM-dissected epidermis from PP skin and LCM-dissected epidermis from PN skin (n = 3). (C) Expression of AP-1 component genes was evaluated in LCM-dissected dermis from PP skin and LCM-dissected dermis from PN skin (n = 3). In (B) and (C), asterisk symbols denote genes with significantly altered expression (P < 0.05). In (A) – (C), red labels denote genes with significantly elevated expression in lesional skin, while blue labels denote genes with significantly decreased expression in lesional skin (P < 0.05). [file 1471-2164-14-527-S10.tiff]

(A)

Average Fold-Change (PP/PN)

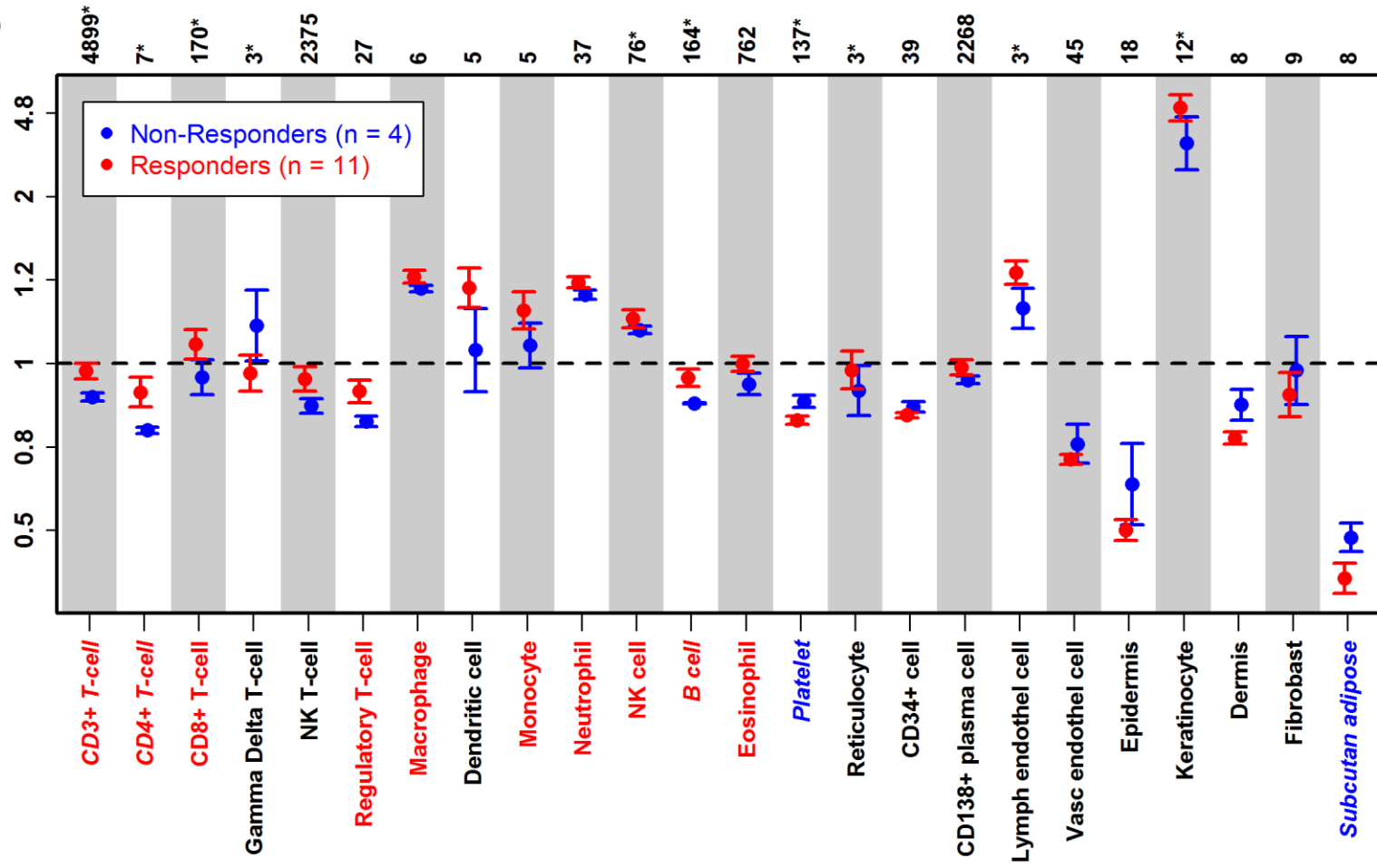

(B)

Average Fold-Change (PP/PN)

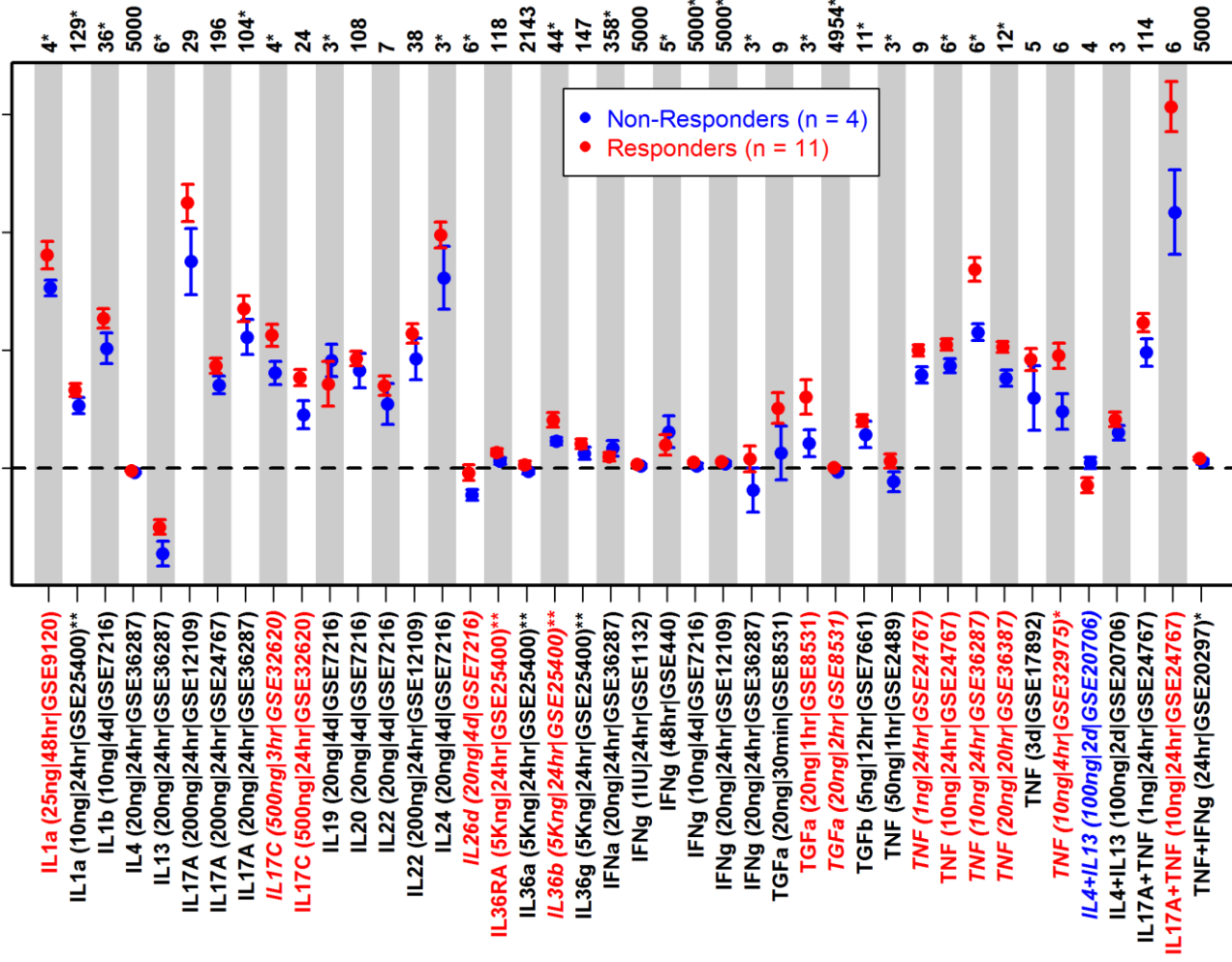

(C)

## Average Fold-Change (PP/PN)

0.707 1 1.414

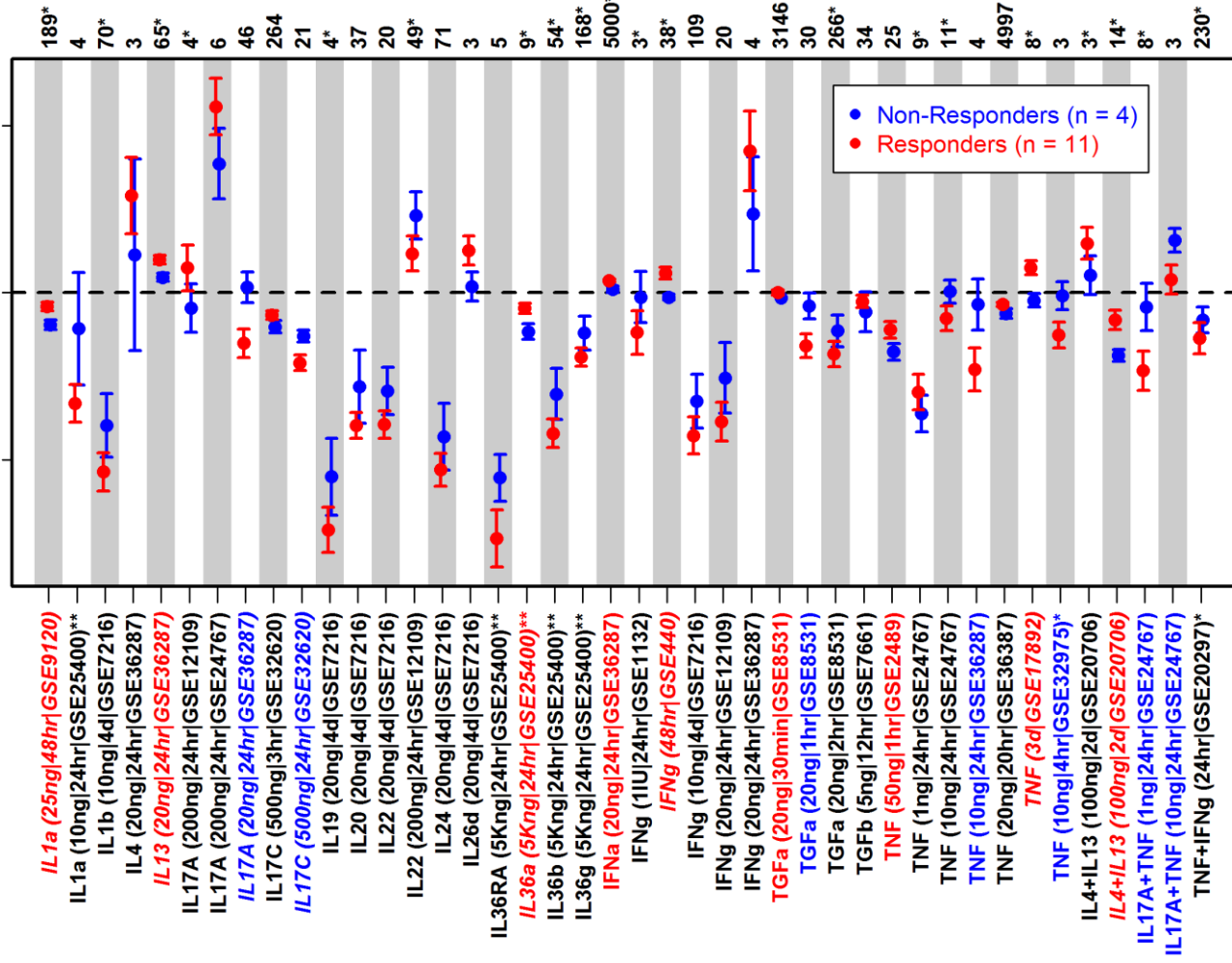

Supplement: Additional file 11 — Identification of inflammatory and cytokine signatures that distinguish etanercept responders from non-responders. (A) For each cell population, signature scores were calculated as the weighted average of fold-changes (PP/PN) among the top N cell type-specific genes (weighted arithmetic mean). The value of N is listed in the top margin for each cell type, and was chosen by searching for values (3 ≤ N ≤ 5000) that maximized separation between responders and non-responders (i.e., minimized the p-value obtained from a two-sample t-test). In signature calculations, genes were weighted according to the square root of their rank (see Methods). Genes were ranked either by p-values generated from the test for cell type-specific expression (asterisk; top margin), or were ranked by the fold-change ratio of a gene’s expression in a given cell type relative to the 23 other cell types (no asterisk; top margin). The ranking approach used for a given threshold was the one leading to better separation between responders and non-responders. Red labels indicate cell populations for which signature scores of responders were at least marginally higher than those of non-responders (P < 0.10; two-sample t-test). Conversely, blue labels denote cell types for which signature scores of non-responders were at least marginally higher than those of responders (P < 0.10; two-sample t-test). Italicized labels denote cases in which signature scores for responders and non-responders differed significantly (P < 0.05). In parts (B) and (C), the same analyses were performed, except signature scores were calculated based upon (B) the N genes most strongly induced in each of 42 cytokine experiments or (C) the N genes most strongly repressed in each of 42 cytokine experiments. [file 1471-2164-14-527-S11.pdf]

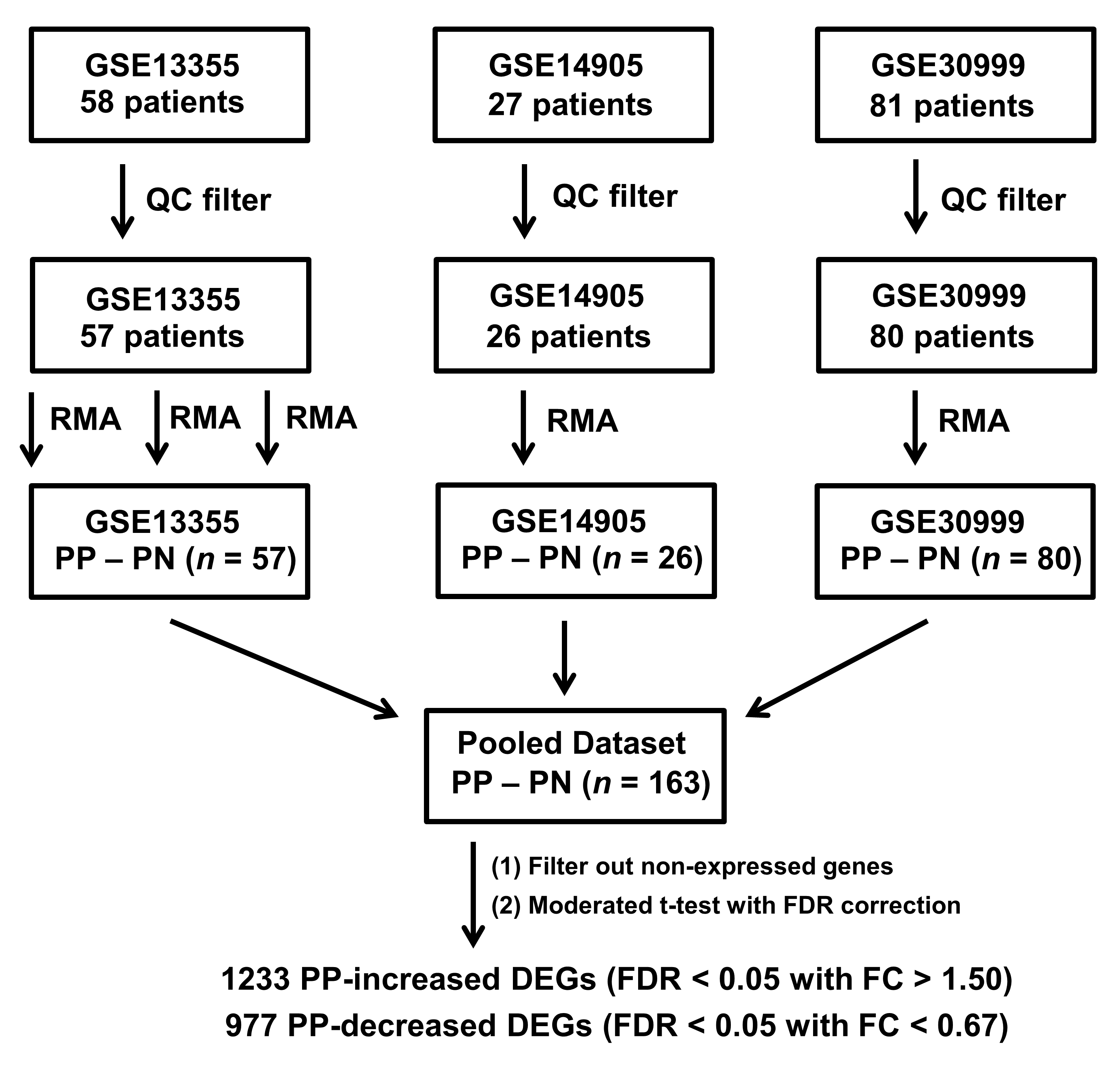

Supplement: Additional file 12 — Overview of data processing steps leading to detection of differentially expressed genes. Paired lesional (PP) and uninvolved (PN) microarray samples generated using the same platform (Affymetrix Human Genome Plus 2.0 array) were obtained from each of three studies (GSE13355, GSE14905 and GSE30999). Following quality control (QC) filtering, CEL files from each study were normalized using robust multichip average (RMA). Samples from GSE13355 were collected in three batches and each batch was normalized separately. Paired PP – PN differences were next calculated for all genes within each dataset, and these differences were subsequently pooled. For each gene, this yielded PP – PN expression differences from 163 patients. We removed from consideration 1233 genes not significantly expressed above background in any of the PP and PN samples. For the remaining 18793 genes, we tested whether the mean PP – PN expression difference (log2 scale) was significantly different from zero (moderated t-test). This led to the identification of 1233 PP-increased DEGs (FDR < 0.05 and FC > 1.50) and 977 PP-decreased DEGs (FDR < 0.05 and FC < 0.67). [file 1471-2164-14-527-S12.tiff]
