# Supplementary material for: Dissecting the psoriasis transcriptome: inflammatory- and cytokine-driven gene expression in lesions from 163 patients
Source: BMC Genomics. 2013 Aug 1;14:527. doi: 10.1186/1471-2164-14-527 (PMC3751090; doi:10.1186/1471-2164-14-527)
Supplement: Additional file 3 — List of 42 cytokine experiments (primary monolayer KC cultures, HaCaT KCs or 3-D reconstituted epidermis). The table lists experiments in which gene expression responses were evaluated in cytokine-treated cells. The label for each experiment indicates the cytokine used, the concentration (per mL), the length of time cells were treated, and the Gene Expression Omnibus accession under which raw data can be accessed. The third column lists the microarray platform used to evaluate gene expression in each experiment and the corresponding Gene Expression Omnibus platform identification number. Further details on each experiment are available from Gene Expression Omnibus or the reference listed in the final column. [file 1471-2164-14-527-S3.pdf]

Additional File 3. List of 42 cytokine experiments (primary monolayer KC cultures, HaCaT KCs or 3-D reconstituted epidermis). The table lists experiments in which gene expression responses were evaluated in cytokine-treated cells. The label for each experiment indicates the cytokine used, the concentration (per mL), the length of time cells were treated, and the Gene Expression Omnibus accession under which raw data can be accessed. The third column lists the microarray platform used to evaluate gene expression in each experiment and the corresponding Gene Expression Omnibus platform identification number. Further details on each experiment are available from Gene Expression Omnibus or the reference listed in the final column.

**Label**	**Cell Type**	**Array Platform**	**Reference**
IL1a (25ng|48hr|GSE9120)	primary KCs	Affymetrix Human Genome U133A 2.0 Array (GPL571)	Yano et al. 2008 (J Cell Physiol 214:1-13)
IL1a (10ng|24hr|GSE25400)	reconsituted epidermis	Affymetrix Human Gene 1.0 ST Array (GPL6244)	Johnston et al. 2011 (J Immunol 186:2613-22)
IL1b (10ng|4d|GSE7216)	primary KCs	Affymetrix Human Genome U133 Plus 2.0 Array (GPL570)	Sa et al. 2007 (J Immunol 178:2229-40)
IL4 (20ng|24hr|GSE36287)	primary KCs	Affymetrix Human Genome U133 Plus 2.0 Array (GPL570)	Swindell et al. 2012 (PLoS ONE 7:e34594)
IL13 (20ng|24hr|GSE36287)	primary KCs	Affymetrix Human Genome U133 Plus 2.0 Array (GPL570)	Swindell et al. 2012 (PLoS ONE 7:e34594)
IL17A (200ng|24hr|GSE12109)	primary KCs	Affymetrix Human Genome U133A 2.0 Array (GPL571)	Nograles et al. 2008 (Br J Dermatol 159:1092-102)
IL17A (200ng|24hr|GSE24767)	primary KCs	Illumina HumanHT-12 V3.0 expression beadchip (GPL6947)	Chiricozzi et al. 2011 (J Invest Dermatol 131:677-87)
IL17A (20ng|24hr|GSE36287)	primary KCs	Affymetrix Human Genome U133 Plus 2.0 Array (GPL570)	Swindell et al. 2012 (PLoS ONE 7:e34594)
IL17C (500ng|3hr|GSE32620)	primary KCs	Agilent-014850 Whole Human Genome Microarray 4x44K G4112F (GPL6480)	Ramirez-Carrozzi et al. 2011 (Nat Immunol 12:1159-66)
IL17C (500ng|24hr|GSE32620)	primary KCs	Agilent-014850 Whole Human Genome Microarray 4x44K G4112F (GPL6480)	Ramirez-Carrozzi et al. 2011 (Nat Immunol 12:1159-66)
IL19 (20ng|4d|GSE7216)	primary KCs	Affymetrix Human Genome U133 Plus 2.0 Array (GPL570)	Sa et al. 2007 (J Immunol 178:2229-40)
IL20 (20ng|4d|GSE7216)	primary KCs	Affymetrix Human Genome U133 Plus 2.0 Array (GPL570)	Sa et al. 2007 (J Immunol 178:2229-40)

IL22 (20ng|4d|GSE7216)	primary KCs	Affymetrix Human Genome U133 Plus 2.0 Array (GPL570)	Sa et al. 2007 (J Immunol 178:2229-40)
IL22 (200ng|24hr|GSE12109)	primary KCs	Affymetrix Human Genome U133A 2.0 Array (GPL571)	Nograles et al. 2008 (Br J Dermatol 159:1092-102)
IL24 (20ng|4d|GSE7216)	primary KCs	Affymetrix Human Genome U133 Plus 2.0 Array (GPL570)	Sa et al. 2007 (J Immunol 178:2229-40)
IL26d (20ng|4d|GSE7216)	primary KCs	Affymetrix Human Genome U133 Plus 2.0 Array (GPL570)	Sa et al. 2007 (J Immunol 178:2229-40)
IL36RA (5Kng|24hr|GSE25400)	reconsituted epidermis	Affymetrix Human Gene 1.0 ST Array (GPL6244)	Johnston et al. 2011 (J Immunol 186:2613-22)
IL36a (5Kng|24hr|GSE25400)	reconsituted epidermis	Affymetrix Human Gene 1.0 ST Array (GPL6244)	Johnston et al. 2011 (J Immunol 186:2613-22)
IL36b (5Kng|24hr|GSE25400)	reconsituted epidermis	Affymetrix Human Gene 1.0 ST Array (GPL6244)	Johnston et al. 2011 (J Immunol 186:2613-22)
IL36g (5Kng|24hr|GSE25400)	reconsituted epidermis	Affymetrix Human Gene 1.0 ST Array (GPL6244)	Johnston et al. 2011 (J Immunol 186:2613-22)
IFNa (20ng|24hr|GSE36287)	primary KCs	Affymetrix Human Genome U133 Plus 2.0 Array (GPL570)	Swindell et al. 2012 (PLoS ONE 7:e34594)
IFNg (1IU|24hr|GSE1132)	primary KCs	Affymetrix Human Genome U133A/U133B Array (GPL96/GPL97)	Mansourian et al. 2004 (Bioinformatics 20:2726-37)
IFNg (48hr|GSE440)	primary KCs	Affymetrix Human Genome U95 Version 2 Array (GPL8300)	Banno et al. 2003 (Antivir Ther 8:541-54)
IFNg (10ng|4d|GSE7216)	primary KCs	Affymetrix Human Genome U133 Plus 2.0 Array (GPL570)	Sa et al. 2007 (J Immunol 178:2229-40)
IFNg (20ng|24hr|GSE12109)	primary KCs	Affymetrix Human Genome U133A 2.0 Array (GPL571)	Nograles et al. 2008 (Br J Dermatol 159:1092-102)
IFNg (20ng|24hr|GSE36287)	primary KCs	Affymetrix Human Genome U133 Plus 2.0 Array (GPL570)	Swindell et al. 2012 (PLoS ONE 7:e34594)
TGFa (20ng|30min|GSE8531)	primary KCs	Affymetrix Human Genome U133A 2.0 Array (GPL571)	Cheng et al. 2008 (J Invest Dermatol 128:1981-90)
TGFa (20ng|1hr|GSE8531)	primary KCs	Affymetrix Human Genome U133A 2.0 Array (GPL571)	Cheng et al. 2008 (J Invest Dermatol 128:1981-90)

TGFa (20ng|2hr|GSE8531)	primary KCs	Affymetrix Human Genome U133A 2.0 Array (GPL571)	Cheng et al. 2008 (J Invest Dermatol 128:1981-90)
TGFb (5ng|12hr|GSE7661)	HaCatT KCs	UHN-Toronto 19K single spotted Human EST microarray v7 (GPL3515)	N/A
TNF (50ng|1hr|GSE2489)	primary KCs	Affymetrix Human Genome U95 Version 2 Array (GPL8300)	Banno et al. 2005 (J Biol Chem 280:18973-80)
TNF (1ng|24hr|GSE24767)	primary KCs	Illumina HumanHT-12 V3.0 expression beadchip (GPL6947)	Chiricozzi et al. 2011 (J Invest Dermatol 131:677-87)
TNF (10ng|24hr|GSE24767)	primary KCs	Illumina HumanHT-12 V3.0 expression beadchip (GPL6947)	Chiricozzi et al. 2011 (J Invest Dermatol 131:677-87)
TNF (10ng|24hr|GSE36287)	primary KCs	Affymetrix Human Genome U133 Plus 2.0 Array (GPL570)	Swindell et al. 2012 (PLoS ONE 7:e34594)
TNF (20ng|20hr|GSE36387)	primary KCs	Affymetrix Human Genome U133 Plus 2.0 Array (GPL570)	Swindell et al. 2012 (PLoS ONE 7:e34594)
TNF (3d|GSE17892)	primary KCs	Homo sapiens 4.8K 02-01 amplified cDNA (GPL1930)	Boccardo et al. 2010 (Carcinogenesis 31:521-31)
TNF (10ng|4hr|GSE32975)	HaCaT KCs	Affymetrix Human Genome U133 Plus 2.0 Array (GPL570)	Fertig et al. 2012 (BMC Genomics 13:160)
IL4+IL13 (100ng|2d|GSE20706)	primary KCs	Agilent-014850 Whole Human Genome Microarray 4x44K G4112F (GPL6480)	Hirakawa et al. 2011 (J Immunol 186:4762-70)
IL4+IL13 (100ng|2d|GSE20706)	primary KCs	Agilent-014850 Whole Human Genome Microarray 4x44K G4112F (GPL6480)	Hirakawa et al. 2011 (J Immunol 186:4762-70)
IL17A+TNF (1ng|24hr|GSE24767)	primary KCs	Illumina HumanHT-12 V3.0 expression beadchip (GPL6947)	Chiricozzi et al. 2011 (J Invest Dermatol 131:677-87)
IL17A+TNF (10ng|24hr|GSE24767)	primary KCs	Illumina HumanHT-12 V3.0 expression beadchip (GPL6947)	Chiricozzi et al. 2011 (J Invest Dermatol 131:677-87)
TNF+IFNg (24hr|GSE20297)	HaCaT KCs	Affymetrix Human Genome U133 Plus 2.0 Array (GPL570)	Fujita et al. 2011 (J Invest Dermatol 131:1660-7)
